# A pilot study on the impact mechanism of internal and external leading variables on consumers’ purchase intention and healthy dietary behavior of plant-rich foods

**DOI:** 10.1371/journal.pone.0353756

**Published:** 2026-07-16

**Authors:** Limin Fang, Shahriman Zainal Abidin, Lesi Zhou, Juncheng Mu, Honglei Lu

**Affiliations:** 1 Faculty of Art & Ceramic Design, Jingdezhen University, Jingdezhen, China; 2 Faculty of Art & Design, Universiti Teknologi MARA, Shah Alam, Selangor Darul Ehsan, Malaysia; 3 School of Fine Arts, Nanjing Normal University, Nanjing, China; 4 School of Art, ANHUI University, Hefei, China; Sreenidhi Institute of Science and Technology, INDIA

## Abstract

This study integrates the TPB with Information Processing Theory and Sensory Marketing Theory to investigate the influence mechanisms of Plant-Rich Foods(PRF) attributes and their packaging on consumer purchase intention and healthy eating behaviors. Through the construction of a structural equation model, empirical analysis was conducted on seven core variables and their interrelationships: consumer attitude(CA), socio-cultural environment(SE), consumer individual requirements(CIR), packaging environmental considerations(ECP), perceived experiential value(PEV), food information factors(FIF), and packaging functional attributes(FPP), thereby validating the proposed hypotheses. The results indicate that all seven variables significantly and positively influence purchase intention, albeit with varying strengths. Packaging functional attributes demonstrated the strongest driving force, followed by individual consumer needs and food information factors. Perceived experiential value, consumer attitude, and packaging environmental considerations exhibited moderate influence, while the socio-cultural environment exerted the weakest impact. The overall influence of “externally oriented” product variables on purchase intention surpassed that of “internally oriented” consumer variables. The impact on healthy eating behaviors presented a dual logic of “direct drive and indirect transmission.” consumer individual requirements exhibited a weaker direct influence on healthy eating behaviors compared to their influence on purchase intention, forming a chained transmission pathway from individual needs to purchase intention to healthy behaviors. Theoretically, this research extends the application of the Theory of Planned Behavior, elucidates the transmission mechanisms of variables, and constructs a multidimensional relational framework. Practically, it offers direction for plant-based food enterprises in optimizing packaging and marketing communication strategies, and provides a reference basis for policymakers.

## Introduction

In the contemporary landscape of health-conscious lifestyles and sustainable development, Plant-Rich Foods (PRF) are gaining unprecedented attention, becoming a focal point in global dietary research and consumer markets [1]. PRF encompasses a variety of foods including vegetables, fruits, whole grains, legumes, nuts, and seeds. These foods are rich in dietary fiber, vitamins, minerals, plant-based proteins, and polyphenols, all of which are crucial for human health. They also contain unique bioactive compounds such as phytosterols and isoflavones, which work synergistically to fortify human health [1,2].

Scientific research has revealed a close relationship between PRF and human health. Diets primarily based on PRF can effectively reduce the risk of chronic diseases such as cardiovascular diseases, diabetes, obesity, and various cancers [3]. For example, dietary fiber in PRF can regulate gut microbiota and maintain gut health, thereby influencing overall metabolism [4]. Plant polyphenols possess potent antioxidant and anti-inflammatory properties, which help protect cells from oxidative stress and inflammatory damage, playing a positive role in preventing cardiovascular diseases and delaying aging [5]. Simultaneously, PRF plays a significant role in environmental protection. Compared to animal-based foods, the production of PRF requires less land, water resources, and energy, and results in lower greenhouse gas emissions, making it a key component of sustainable diets [6].

Currently, the PRF-related industry is experiencing vigorous development. The global plant-based food market continues to expand, with innovative products emerging constantly. These range from plant-based protein beverages and plant-based meat products to functional foods rich in plant ingredients, catering to the diverse needs of consumers [1]. The main classifications and core nutritional elements of PRF indicate that PRF centers on plant-based ingredients, including natural plant foods (vegetables, fruits, etc.) and plant-based dietary supplements [7]. It is evident that PRF reduces the risk of chronic diseases through multiple mechanisms (nutrient supply, gut microbiota regulation, and antioxidant effects), and its health benefits have been supported in the fields of cardiovascular health, metabolism, and oncology [8]. However, in reality, consumers often lack sufficient understanding of PRF foods. Research indicates that in the United States, consumer acceptance of plant-based foods is relatively low. Despite high awareness of health, environmental, and social sustainability, less than half of consumers change their purchasing behavior accordingly [9]. Similar situations exist in other countries and regions. This phenomenon is largely due to the perception that while plant-based foods are healthier than animal-based foods, there are varying levels of understanding regarding their nutritional value and health benefits [9]. Based on these research findings, it is clear that there are certain barriers to consumer understanding of PRF foods. Consumers are hesitant to try plant-based foods, primarily due to a lack of confidence in their nutritional value and health benefits [9]. Furthermore, consumer understanding of the environmental impact of plant-based foods is often insufficient, largely stemming from information presented on food labels and packaging [10,11]. Therefore, in the face of societal skepticism regarding Plant-Rich Foods, it is crucial to further investigate the impact of PRF food packaging attributes on consumer behavior.

The organic integration of the Theory of Planned Behavior (TPB), Information Processing Theory, and Sensory Marketing Theory is fundamentally rooted in their multidimensional complementarity in explaining consumer behavioral decision-making. This integration aligns perfectly with the core appeals of research into PRF (Plant-rich Foods) and their packaging. As a special food category rich in plant-based ingredients, the consumption decisions for PRF are driven by rational factors like health perceptions and environmental values, as well as by hedonic factors such as taste expectations and sensory experiences. A single theory is insufficient to encompass this complex process. TPB focuses on the internal psychological motivations of “attitude-subjective norm-perceived behavioral control” [12], which precisely corresponds to key psychological variables in PRF consumption, such as consumers’ attitudes towards health benefits, societal advocacy for sustainable diets, and the perceived ease of trying plant-based foods. However, TPB lacks a clear explanation of “how packaging influences these variables.” Information Processing Theory, with packaging as the central information carrier [13], provides a targeted rational cognitive basis for the formation of attitudes in TPB. The health information it conveys (e.g., plant protein, dietary fiber content), environmental labels (e.g., biodegradable material descriptions), and social cues (e.g., “recommended for sustainable diets”) directly address the critical pain point of “insufficient cognition” in PRF consumption, shaping consumers’ judgments of PRF’s nutritional and social value. Sensory Marketing Theory [14], on the other hand, utilizes sensory stimuli such as visual cues (natural color palettes, presentation of ingredient textures) and tactile sensations (texture of natural materials) to evoke positive emotions. This injects emotional momentum into TPB variables, thereby strengthening positive attitudes towards PRF and reducing psychological resistance and consumption barriers associated with “plants equaling unpleasant taste.”

This integrated framework offers strong targeted explanatory power and practical value for research on PRF foods and their packaging. To address the core barrier of “vague consumer perception of nutritional value” in PRF consumption, clear packaging information (e.g., quantified nutritional content, concise health benefits) fills cognitive gaps through cognitive processing, activating positive attitudes. This is consistent with findings in plant-based food labeling research indicating that “nutritional claims such as vitamins and proteins can significantly increase selection probability” [15]. To tackle the emotional resistance of “poor taste of plant-based foods,” the sensory design of PRF packaging (e.g., visual presentation mimicking natural ingredients, warm tactile textures) evokes pleasure and trust, effectively mitigating biases and strengthening attitudes and perceived behavioral control [16]. As related research confirms, multi-sensory contextual cues can significantly improve consumers’ taste perception and willingness to choose plant-based products [15,17]. To counter behavioral inertia stemming from “inconvenience and complex decision-making,” practical guidance from Information Processing (e.g., cooking suggestions, pairing options) and convenient experiences designed by Sensory Marketing (e.g., easy-tear openings, portable formats) collectively enhance perceived behavioral control. Combined with subjective norms activated by social cues conveyed by packaging (e.g., “a choice for health enthusiasts”), this drives the transformation of behavioral intentions, echoing the conclusion in TPB’s application to food consumption research that “attitude, subjective norm, and perceived behavioral control jointly drive purchase intention” [12]. On a theoretical level, this integration enriches the application of the “external stimuli-internal decision” conversion mechanism in PRF packaging research, constructing a multidimensional analytical framework that spans cognition, emotion, and motivation, thereby addressing the deficiency of “lack of multidimensional interaction analysis” in PRF packaging research. On a practical level, it clarifies the directions for information optimization (e.g., simplifying jargon, highlighting core nutrition) and sensory design (e.g., application of natural materials, scenario-based visual presentation) for PRF packaging. This not only provides the underlying logical explanation for “why some PRF products, despite their health value, have low penetration rates” but also offers a clear research path for PRF packaging studies: “theoretical integration-variable correlation-empirical testing.” Simultaneously, it provides operational strategic support for businesses to optimize PRF packaging design, enhance product market acceptance, and promote consumers’ healthy dietary behavior change, achieving a unified balance between theoretical explanatory power and practical guidance for PRF research.

This paper integrates the Theory of Planned Behavior (TPB) [18], information processing theory [19], and sensory marketing theory [20] to construct a theoretical framework for how Plant-Rich Foods (PRF) and their packaging factors promote consumers’ intentions toward healthy eating behaviors. Within the TPB, consumers’ behavioral intentions are jointly determined by attitudes (consumers’ health perceptions of PRF packaging) [21], subjective norms (the influence of the social environment on PRF) [22], and perceived behavioral control (new consumers’ personal needs) [23]. In information processing theory, packaging serves as an information carrier, and its design factors (such as environmentally friendly packaging, health information, and food information) [24]influence consumers’ cognitive processing of PRF’s health attributes. In sensory marketing theory, sensory stimuli such as the visual and tactile aspects of packaging (perceived experiential value) [25] can evoke emotional responses, thereby affecting consumers’ behavioral intentions. Combining these theories with the influence of Plant-Rich Foods (PRF) packaging on consumer behavior, the internal factors of the Theory of Planned Behavior (TPB), namely attitudes (consumers’ health perceptions of PRF packaging), subjective norms (the influence of the social environment on PRF), and perceived behavioral control (consumers’ personal needs), are used to explore the behavioral impact on consumers’ purchase intentions and healthy eating behaviors. Simultaneously, information processing (such as environmentally friendly packaging, health information, and food information) and sensory marketing (perceived experiential value) in packaging factors are considered as external factors to explore their impact on consumers’ purchase intentions and healthy eating behaviors. The complementary nature of the three assumptions—balancing rational decision-making with irrational emotions—precisely matches the dual functions of PRF packaging in “information delivery and experience creation.” Together, they construct a mechanism through which external stimuli from PRF packaging influence consumers’ actual internal needs, forming a dual-path synergy of “cognitive foundation and emotional empowerment.” This allows the abstract psychological variables of TPB (Theory of Planned Behavior) to gain clear external activation pathways within the PRF context, overcoming the explanatory limitations of single theories in PRF consumption research.

Based on this, the purpose of this study is to reveal the mechanism of PRF packaging on healthy eating behaviors. By integrating the Theory of Planned Behavior, information processing theory, and sensory marketing theory, this study systematically analyzes how the design elements of PRF packaging (such as information transmission and sensory experience) influence consumers’ purchase intentions and healthy eating behavior choices through three levels: cognitive processing, emotional arousal, and psychological motivation. At the same time, this study constructs a multi-dimensional theoretical explanation framework, breaking through the limitations of a single theoretical perspective. It organically combines the internal psychological drivers of TPB, the cognitive logic of information processing, and the emotional triggering mechanism of sensory marketing, forming a complete logical chain of “cognition – emotion – intention – behavior,” providing interdisciplinary theoretical support for PRF food packaging design. In addition, this study aims to optimize the practice of PRF packaging. By clarifying the key influencing factors of packaging design (such as the clarity of health information and the texture of sensory experience) through the theoretical framework, it provides actionable strategic directions for enterprises to improve the market penetration of PRF products and promote the transformation of consumers’ healthy eating behaviors.

Meanwhile, this study complements and innovates on existing research. Traditional research often uses TPB alone to analyze consumer attitudes and intentions, ignoring the impact of packaging information design on cognitive processes; or it only explores experiential value from the perspective of sensory marketing, lacking consideration of psychological factors such as social norms and self-control. In response to these shortcomings, this study integrates multiple theories, linking internal psychological variables of “attitude – subjective norms – perceived behavioral control” with external environmental variables of “information processing – sensory stimuli,” filling the gap in the explanation of the “external stimuli – internal decision” transformation mechanism by a single theory. Furthermore, in the field of PRF packaging design, most studies focus on the impact of a single attribute of packaging (such as environmental friendliness or visual design) on consumer behavior, lacking a multi-dimensional interactive analysis of information transmission, sensory experience, and psychological motivation. This study clarifies how information processing factors (such as food information) and sensory marketing factors (such as tactile experience) in packaging indirectly affect healthy eating behavior intentions by influencing variables such as attitudes and subjective norms in TPB, constructing a theoretical model of multi-variable synergistic influence.

Therefore, this study can enhance consumer awareness of plant-based ready-to-eat (PRF) food by improving PRF food packaging design, particularly regarding its nutritional value and health benefits. Simultaneously, it is crucial to strengthen consumer education and promotion of PRF foods to increase their understanding and acceptance of the PRF food concept. Food companies can meet consumer demands and expectations for plant-based foods by improving product design and increasing transparency. Furthermore, packaging serves as a “silent salesperson,” directly addressing consumer barriers. It attempts to reduce the difficulty of using PRF through packaging cognition while eliminating the consumer bias that “plant-based = unpalatable” through sensory experiences. This is achieved by communicating the health benefits of PRF food and the concept of a healthy lifestyle through PRF food packaging.

Despite the widely acknowledged health and environmental value of plant-based foods, consumers still face cognitive barriers and exhibit low purchase conversion rates. Existing research predominantly employs the Theory of Planned Behavior (TPB) to explain consumer attitudes, examines packaging experience solely from a sensory marketing perspective, or investigates consumer purchase decision-making behavior under a singular dimension [26–28]. These studies overlook the intervening role of packaging information design in cognitive processes and fail to comprehensively consider psychological factors such as social norms and self-control. Furthermore, the systematic influence of packaging’s multi-dimensional attributes (functional, informational, eco-friendly, experiential) on purchase intention and healthy eating behaviors, mediated by the synergistic effects of internal and external variables, has not been fully elucidated. Therefore, this study integrates multiple theoretical perspectives to address the core question: How do internal and external leading variables of plant-based food packaging influence consumers’ purchase intention and healthy eating behaviors? What are the structural characteristics of their influence pathways and strengths?

The innovation of this research lies in three aspects: First, it transcends the limitations of TPB, which primarily focuses on internal psychological variables, by innovatively integrating Information Processing Theory and Sensory Marketing Theory to construct a sequential mediation model of “Internal Psychological Factors – External Stimuli – Behavioral Intention – Healthy Behaviors.” This elevates TPB from a “psychologically driven model” to a “psychologically and product-driven model.” Second, by quantifying and comparing the path coefficients of various variables, it uniquely reveals the “hierarchical driving characteristics” of core variables on purchase intention and the “path differentiation logic” influencing healthy eating behaviors, challenging the notion of homogeneous effects of variables on intentions and behaviors. Third, it constructs a multidimensional association framework comprising “core internal factors of TPB + external driving factors of product packaging,” thereby addressing the issue of abstract conceptualization within TPB’s core constructs and providing a reusable paradigm for interdisciplinary research.

The subsequent structure of this paper is organized as follows: Section II presents a comprehensive literature review, systematically examining the theoretical underpinnings of TPB, plant-based food packaging factors, and internal and external variables. Section III proposes research hypotheses and the theoretical model. Section IV details the questionnaire design and research methodology, including sample selection, measurement instruments, and data analysis techniques. Section V reports the empirical research results, encompassing sample characteristics, reliability and validity tests, and hypothesis testing. Section VI provides a discussion, offering an in-depth analysis integrating theoretical and empirical findings. Section VII elaborates on the theoretical contributions and practical implications. Section VIII highlights the research limitations and future directions, followed by the conclusion.

## Literature review

### Theory of planned behavior

The Theory of Planned Behavior (TPB), introduced by Ajzen, is a prominent theoretical model in behavioral science used to predict and explain human behavior. The core tenets of the theory include: Behavioral Beliefs, which are an individual’s beliefs about the behavior itself and its outcomes, influencing their attitude toward the behavior; Attitude toward the Behavior, which is an individual’s positive or negative evaluation of the behavior, based on behavioral beliefs and outcome evaluations; Normative Beliefs, which represent an individual’s perception of how others view their behavior, reflecting social expectations; Subjective Norm, which is an individual’s perception of the expectations of important others regarding their behavior, and their willingness to comply with these expectations; Perceived Behavioral Control, which refers to an individual’s perception of their ability and resources to perform a specific behavior; Behavioral Intention, which is an individual’s motivation to perform a specific behavior, determined by attitude, subjective norm, and perceived behavioral control; and Behavior, where behavioral intention is the direct determinant, which in turn influences actual behavior [12]. Based on these components, the TPB emphasizes that an individual’s behavior is primarily determined by behavioral intention, which is influenced by attitude, subjective norm, and perceived behavioral control [12]. This theory has been widely applied and validated across various fields, particularly in health behavior and consumer behavior [29].

Research indicates that the TPB has been extensively used to explain and predict consumer purchasing behavior regarding environmentally friendly products, such as organic foods or energy-efficient products [30]. It is also widely applied in consumers’ actual health behaviors, such as dietary habits and exercise intentions and behaviors [31]. In addition to its broad application in consumer behavior, the TPB has also been applied in the field of food packaging’s influence on consumer behavior. Studies have found that the TPB can be used to explore the behavioral impact of food packaging on consumer behavior [32]. For example, sustainable packaging in the food industry significantly enhances consumers’ purchase intentions [33]. Furthermore, extended TPB models can effectively predict consumer purchase intentions under intelligent packaging [34]. Therefore, the TPB can serve as a structured framework for research predicting the impact of PRF food on consumer behavior.

### PRF food packaging factors

Plant-Rich Foods (PRF) present unique requirements for food packaging. As PRF primarily consists of plant-based foods, including vegetables, fruits, whole grains, legumes, nuts, seeds, and plant oils [2], these specific needs are mainly reflected in the functionality and environmental friendliness of the packaging. For instance, in terms of functionality, the materials must possess excellent gas barrier properties to control oxygen and carbon dioxide levels within the package, thereby extending the shelf life and preventing spoilage [35]. Simultaneously, the packaging materials should have appropriate moisture permeability to accommodate the respiration and transpiration needs of different foods [35]. Therefore, what distinguishes PRF packaging from that of other foods is that, in addition to ensuring food safety and quality [36], it also requires specialized functional design based on the preservation needs of these foods. Environmental friendliness is also a crucial factor in PRF food packaging. Research indicates that an increasing number of bio-based materials (such as PLA, PCL, PBSA, etc.) are being used to replace traditional plastics to reduce plastic pollution and resource consumption. Recyclable and biodegradable packaging materials (such as PCR-PP) are also being gradually promoted to align with sustainable development trends. Consequently, with advancements in materials science and the growing popularity of the healthy lifestyle associated with PRF foods, environmentally friendly packaging design aligns with sustainable development trends and the healthy lifestyle promoted by these foods. Furthermore, in terms of consumer preferences, it is essential to consider the actual consumer experience. Consumer preferences for packaging include shape and material, the quantity of packaged products, opening and sealing methods, storage capacity, and the volume of each food item [37]. In addition, information on health, transparency, and authenticity in packaging influences consumer intentions, such as the use of traceable materials and the emphasis on the health benefits of the product [38]. Therefore, packaging can enhance consumer behavioral intentions through clear information.

### Internal psychological variables and external environmental variables

This study extends and innovates upon existing theoretical applications by integrating Plant-Rich Food (PRF) and its packaging elements. Traditional research often employs the Theory of Planned Behavior (TPB) in isolation to analyze consumer behavioral intentions, neglecting the impact of packaging information design on cognitive processes. Alternatively, it may explore experiential value solely from a sensory marketing perspective, lacking consideration for psychological factors such as social norms and self-control. Addressing these limitations, this research builds upon the core logic of multiple theories, first clarifying the connotations and theoretical underpinnings of the core variables. Internal psychological variables, derived from the core constructs of TPB theory, refer to the intrinsic psychological mechanisms driving consumer decisions. These include: Attitudes, defined according to TPB theory as consumers’ sustained evaluative tendencies towards PRFs and their associated practices (healthy eating behaviors) [12]. In this study, it specifically refers to consumers’ comprehensive judgment of the “health value-environmental value-edible value” of PRF foods through packaging information and sensory experiences. Its theoretical basis can be traced back to Attitude Functions Theory [39], which posits that attitudes are the integration of an individual’s cognitive and affective responses towards an object. The information and sensory stimuli from PRF packaging serve as critical input sources for attitude formation. Subjective Norms, defined according to TPB theory as consumers’ perceived “expectations and pressures from important others (family, friends, health experts) or social groups (sustainable eating communities) regarding their choice of PRFs” [12]. Its theoretical foundation stems from Social Influence Theory [40], which suggests that social norms influence individual decisions through informational influence (practical information provided by others’ behaviors) and normative influence (pressure to conform to group expectations). Cues such as “social recommendations” and “group choices” conveyed by PRF packaging elements act as significant external triggers for subjective norms. Perceived Behavioral Control, defined according to TPB theory as consumers’ perception of “whether they have the ability and resources to implement PRF healthy eating behaviors” [12]. Its theoretical basis comes from Self-Efficacy Theory [41], which states that an individual’s judgment of the difficulty of a behavior directly affects their behavioral intentions. The convenient design of PRF packaging (e.g., easy-open tabs, portable sizes) and informational guidance (e.g., nutritional facts, preparation instructions) are core carriers for reducing behavioral difficulty and enhancing perceived control. External environmental variables integrate core elements from Information Processing Theory and Sensory Marketing Theory, acting as external stimuli conveyed through PRF packaging that influence consumers’ cognition and affect. These include: Information Processing Factors, which, according to Information Processing Theory [13], are the key contents conveyed by PRF packaging as an information carrier that influence consumers’ cognitive processing. This encompasses health information descriptions (quantified nutritional component tables), food information descriptions (e.g., ingredient sourcing, preparation methods, shelf life), and environmental information descriptions (e.g., biodegradable material labels, carbon footprint data). Consumers’ core cognitive gaps in PRF consumption (nutritional value, environmental value) can be effectively bridged by packaging information. Sensory Marketing Factors, according to Sensory Marketing Theory [14], are the stimuli conveyed by PRF packaging through sensory channels such as vision (color, shape, texture) and touch (material texture, weight). The core objective is to evoke consumers’ perceived experiential value (pleasure, trust, naturalness). Its theoretical basis lies in Embodied Cognition Theory [42], which posits that sensory experiences directly influence an individual’s affective and cognitive judgments. For instance, the tactile sensation of natural bamboo pulp material and the visual stimulation of fresh green colors on PRF packaging can evoke emotional associations of “health and naturalness” through embodied mechanisms, thereby mitigating resistance to plant-based foods.

Leveraging the clearly defined variables and theoretical underpinnings previously established, this study integrates multiple theories to conduct a linked analysis of internal psychological variables—“attitude-subjective norm-perceived behavioral control”—and external environmental variables—“information processing-sensory stimuli.” This approach aims to bridge the explanatory gap left by single theories regarding the conversion mechanism of “external stimuli to internal decision-making.” Traditional TPB research has not explicitly detailed the external trigger pathways for internal psychological variables. However, by delineating specific dimensions of packaging information and sensory stimuli, this research clarifies how external variables influence consumers internally through cognitive processing (information processing) and emotional arousal (sensory marketing), thereby providing clear theoretical logic for the “external stimuli-internal decision-making” transition. Furthermore, within the domain of PRF packaging design, the majority of research has concentrated on the impact of singular packaging attributes (such as environmental friendliness or visual design) on consumer behavior, lacking an analysis of the multidimensional interaction between information transmission, sensory experience, and psychological motivation. This study, grounded in the precise definition of variables and robust theoretical foundations, elucidates how information processing factors in packaging (e.g., food information labels) influence consumers’ perceptions of PRF. Concurrently, sensory marketing factors, through their inherent cognitive mechanisms, evoke positive consumer emotions, attitudes, and perceived behavioral control, ultimately impacting consumers’ intentions for healthy eating behavior. This culminates in the construction of a multivariate synergistic influence theoretical model: “external environmental variables-internal psychological variables-behavioral intention.” This model not only addresses the shortcomings of “ambiguous variable definition and weak theoretical basis” in PRF packaging research but also transcends the limitations of single-attribute studies, rendering the model more theoretically rigorous and practically pertinent.

## Research hypotheses and models

### Research hypothesis

#### Consumer attitudes toward plant-rich foods.

In the Theory of Planned Behavior (TPB), attitude is defined as an individual’s sustained positive or negative evaluation of a target behavior, serving as a core antecedent to behavioral intentions [12]. A consumer’s attitude toward Plant-Rich Foods (PRF) fundamentally represents their composite evaluative tendency, stemming from their cognitions and appraisals of such foods [43]. From a theoretical standpoint, Attitude Functions Theory [39] posits that attitudes form from an individual’s pursuit of “instrumental value (e.g., health benefits) and social value (e.g., environmental identity),” which aligns closely with the core attributes of PRF. Specifically, health value is the primary driver for consumers forming positive attitudes: empirical research indicates that PRF, rich in bioactive compounds like dietary fiber and polyphenols, can mitigate risks of chronic diseases such as diabetes and cardiovascular disease by modulating gut microbiota and suppressing inflammatory responses [1]. A clear perception of health benefits directly shapes consumers’ positive attitudes toward PRF [43]. Secondly, sensory experience and quality perception are crucial complements to attitude formation: consumer acceptance of PRF is highly dependent on sensory experiences like taste and flavor; when PRF products meet expectations for “deliciousness and high quality,” attitudes significantly improve in a positive direction [9]. Furthermore, environmental and economic factors also contribute significantly to attitude formation: environmentally conscious consumers develop positive attitudes due to PRF’s low greenhouse gas emissions and resource consumption during production [44], while cost-effective PRF products (affordable with outstanding nutritional value) are more readily embraced by a broader consumer base [9]. Linking theory and empirical evidence, the core logic of TPB has been substantiated by numerous studies, demonstrating that positive consumer attitudes toward PRF enhance behavioral motivation and significantly increase purchase intentions [45]. The underlying mechanism for this association is that positive attitudes reduce consumers’ psychological resistance (e.g., the bias that “plant-based foods are unpalatable”) while reinforcing the cognition that “choosing PRF is a wise decision,” thereby propelling the formation of purchase intentions. Based on the TPB framework and the aforementioned empirical evidence, this paper proposes the hypothesis

H1: Consumers’ attitudes toward PRF foods have a positive impact on consumers’ intention to purchase PRF foods.

#### The influence of the social environment on plant-rich foods.

According to Ajzen’s Theory of Planned Behavior, subjective norms, defined as “perceived social pressure,” are a core variable influencing behavioral intentions [12]. The social environment serves as the central conduit and trigger for these subjective norms. Social Impact Theory [40] posits that an individual’s behavioral decisions are subject to the dual influence of “significant other influence and group norm pressure,” providing theoretical support for how the social environment impacts purchase intentions for plant-based foods (PRF). Specifically, the social environment influences consumers through three channels: family, reference groups, and socio-cultural factors. At the family level, the dietary preferences and purchasing behaviors of family members create a direct modeling effect. If family members widely choose PRFs, consumers perceive strong “family expectation pressure,” thereby increasing their purchase intention [46]. At the reference group level, recommendations and examples from friends, colleagues, and health experts can reinforce purchase intentions through both “informational influence (providing health/environmental information about PRFs)” and “normative influence (conforming to group identity)” [47]. At the socio-cultural level, with the widespread promotion of concepts like “sustainable diets” and “Healthy China,” consumers perceive increased “social correctness pressure.” Choosing PRFs is seen as behavior aligned with societal expectations, and this recognition of social norms directly translates into purchase intention [48]. The core logic underpinning this perspective is that the social environment, by activating the subjective norm variable within TPB, reduces consumers’ “social risk” in choosing PRFs (e.g., fear of being questioned by others) while strengthening the perception that “choosing PRFs aligns with social expectations,” consequently driving the formation of purchase intention. It is noteworthy that there is a potential interaction between the social environment and consumer attitudes. A positive social environment (e.g., widespread acceptance of PRFs among peers) can bolster a consumer’s positive attitude towards PRFs, and this attitude, in turn, further amplifies the social environment’s influence on purchase intention, forming a “social environment-attitude-purchase intention” pathway. However, this section focuses on the direct impact of the social environment, with its interaction with attitudes to be further elaborated in subsequent models. Based on TPB theory, social impact theory, and empirical evidence, this paper proposes the hypothesis:

H2: The social environment has a positive impact on consumers’ willingness to purchase PRF foods.

#### Consumers’ personal needs for plant-rich foods.

According to the Theory of Planned Behavior (TPB), perceived behavioral control refers to an individual’s perception of “whether they have the ability to perform the target behavior.” Its core essence is intrinsically linked to consumers’ personal needs – consumers’ personal needs are fundamentally a “desire for a state of deficiency” (e.g., a deficiency in health, a lack of environmental identification), and this desire translates into a perceived control of “performing actions to meet needs” [49] Self-Determination Theory further elaborates that when an individual’s core needs (such as health, autonomy, environmental concern) are met, behavioral motivation is significantly strengthened, providing a theoretical basis for how personal needs influence purchase intention and healthy eating behaviors [50]. Consumers’ personal needs for Plant-based Ready-to-eat Foods (PRF) are multi-dimensional, primarily including: health needs (obtaining nutrition and preventing diseases through PRF intake) [51], environmental needs (practicing a sustainable lifestyle by choosing PRF) [52], price needs (pursuing high-value PRF products) [53], and information needs (obtaining transparent information on PRF’s nutritional content, consumption methods, etc.) [54]. Regarding the relationship between personal needs and purchase intention, an extended TPB model has confirmed that perceived behavioral control (whose core driver is the fulfillment of personal needs) is a key variable in predicting purchase intention [12]. Specifically, the satisfaction of health needs can directly strengthen purchase motivation: when consumers perceive that PRF can meet their core need of “disease prevention and health improvement,” they will consider “choosing PRF as feasible and beneficial,” thereby increasing purchase intention [45]. The satisfaction of environmental needs strengthens intention through value congruence: consumers with strong environmental needs will develop a cognition of “behavior aligning with values” due to the sustainable attributes of PRF, promoting the formation of purchase intention [48,53]. The satisfaction of price and information needs enhances intention by reducing perceived barriers: high-value PRF products reduce economic barriers, and transparent information presentation reduces decision-making barriers, both of which enhance perceived behavioral control and, consequently, strengthen purchase intention [54,55]. Based on this, this paper proposes the hypothesis:

H3a: Consumers’ personal needs have a positive impact on their behavioral intentions to purchase PRF foods.

Further examining the relationship between personal needs and healthy eating behaviors, Self-Determination Theory suggests that when an individual’s core needs (such as health and environmental concern) are met through behavior, the behavior shifts from “extrinsic motivation” to “intrinsic motivation,” thereby promoting the formation of sustained behaviors [50]. Specifically, when consumers’ health needs are met through PRF intake (e.g., improved physical condition), the cognition that “PRF offers tangible health benefits” is reinforced, driving the conversion of purchase intention into long-term healthy eating behaviors [56]. The satisfaction of environmental needs strengthens behavioral sustainability through “self-identity”: when consumers perceive that choosing PRF is an act of environmental advocacy, they form the cognition that “healthy eating equals a responsible lifestyle,” thereby promoting the stable formation of healthy eating behaviors [57]. Additionally, personal needs can indirectly influence healthy eating behaviors by moderating the relationship between attitudes and behavioral intentions: when consumers’ personal needs are adequately met, their positive attitudes toward PRF become more stable, and the barriers to converting behavioral intentions into behaviors are reduced [56]. Based on this, this paper proposes the hypothesis:

H3b: Consumers’ personal needs have a positive impact on their formation of healthy eating behavior.

#### Behavioral intentions of consumers to purchase PRF foods.

One of the core propositions of the Theory of Planned Behavior (TPB) posits that “behavioral intention is the most direct antecedent to actual behavior.” The underlying logic is that behavioral intention reflects an individual’s motivational strength and readiness to perform a target behavior, effectively integrating the composite influences of “attitude, subjective norm, and perceived behavioral control,” thereby directly driving the occurrence of behavior [12]. In the realm of Plant-Based Refrigerated Foods (PRF) consumption, this theoretical logic holds true: purchase intention is a consumer’s psychological inclination to “willingly choose and prepare to purchase PRF,” formed after comprehensively considering their attitude towards PRF, societal influences, and personal needs. This inclination, in turn, directly promotes the implementation of healthy eating behaviors by reducing behavioral initiation barriers and clarifying behavioral goals [58].

From a theoretical-empirical linkage perspective, extensive research has validated the positive correlation between PRF purchase intention and healthy eating behaviors. Firstly, from the perspective of health benefits, strengthened purchase intention signifies a firmer consumer perception of PRF’s health value, leading to a greater willingness to incorporate PRF into their daily diet and establish a healthy eating pattern centered around PRF [58]. Secondly, in terms of behavioral sustainability, purchase intention can foster the maintenance of healthy eating behaviors through a “goal setting-self reinforcement” cycle: once consumers form a clear PRF purchase intention, each act of purchasing and consuming PRF reinforces their self-identity as someone who engages in “healthy eating,” thereby promoting the sustained occurrence of the behavior [59]. Finally, considering the synergy between environmental values and healthy behaviors, PRF purchase intention is often accompanied by an endorsement of sustainable diets. This endorsement imbues healthy eating behaviors with greater meaning, thereby enhancing their stability. Studies indicate that consumers who choose PRF based on both health and environmental intentions maintain their healthy eating behaviors significantly longer than those with a single motivation [9]. Furthermore, purchase intention acts as a mediator between “attitude, social environment, and personal needs” and healthy eating behaviors. The positive impact of attitude, the driving force of the social environment, and the motivational role of personal needs all require conversion through purchase intention to effectively influence healthy eating behaviors, forming the core logical chain of this study’s theoretical framework [12,60]. Based on the core propositions of TPB and the aforementioned empirical evidence, this study hypothesizes that:

H4: Consumers’ behavioral intention to purchase PRF foods has a positive impact on their healthy eating behaviors.

#### Impact of external packaging factors on consumers’ behavioral intentions to purchase PRF foods.

The external packaging serves as the primary touchpoint for consumers interacting with Ready-to-Eat (RTE) foods, and its multifaceted attributes significantly influence purchasing intent by deeply affecting consumer behavior. From a perceptual standpoint, the consumption scenarios for RTE foods often involve rapid decision-making environments, where visual presentation and design aesthetics become core variables for capturing attention. Existing research substantiates that visual-tactile linkage factors, such as color saturation, the degree of pictorial representation, and the haptic experience of packaging materials, can directly activate consumers’ sensory pleasure [61]. For RTE foods, fresh and natural color palettes can reinforce associations with “freshness and health,” while ergonomic design enhances expectations of usability. Together, these elements reduce consumers’ decision-making hesitation costs, thereby positively driving purchase intent.

The influence of informational factors stems from consumers’ risk aversion psychology and their need for decision-making information. Consumers of RTE foods generally prioritize product safety, nutritional adequacy, and suitability for their needs. Packaging elements such as product information, brand logos, and nutrition facts labels are critical conduits for mitigating information asymmetry [62]. As a condensation of brand credibility, a well-known brand logo can reduce consumers’ perceived uncertainty about the quality of RTE foods. This brand endorsement is particularly impactful for products with short shelf lives or complex processing, significantly boosting purchase trust. Concurrently, precise and easily understandable nutritional labeling (e.g., calorie count, protein content, allergen warnings) enables consumers to quickly match products with their dietary requirements (e.g., calorie-conscious individuals focusing on calories, fitness enthusiasts on protein). Clear information regarding production date, expiration date, and storage conditions alleviates concerns about “product spoilage.” The completeness and readability of these informational dimensions directly impact consumers’ decision efficiency and purchase intent.

The positive influence of environmental factors is rooted in the current prevalence of green consumption concepts and consumers’ identification with social responsibility. With rising environmental awareness, consumer attention to the eco-friendliness of packaging is continuously increasing. Given that RTE food packaging often includes a substantial proportion of waste (e.g., single-use plastic containers, bags), its environmental attributes are more likely to resonate emotionally with consumers [62]. The use of biodegradable materials (e.g., cornstarch-based plastics, paper packaging), lightweight designs, or packaging with recycling value allows consumers to perceive the brand’s social responsibility, leading to a psychological affirmation that “purchasing aligns with environmental values.” This emotional alignment translates into product preference and purchase intent.

The influence of functional factors centers on the practical value of the packaging and the consumer’s needs within various usage scenarios. The core mechanism is to reduce consumers’ usage costs by enhancing product storage safety, ease of use, and overall experience. The consumption scenarios for RTE foods encompass diverse situations such as home storage, outdoor consumption, and office snacking, requiring packaging functionalities that adapt to the core demands of different settings [63]. For instance, packaging with good sealing properties can extend the shelf life of RTE foods (e.g., ready-to-eat nuts, freeze-dried fruits and vegetables), meeting the long-term storage needs at home. Portable and easy-to-carry packaging designs (e.g., compact, lightweight, spill-proof) are suitable for mobile scenarios like outdoor activities or commuting. Furthermore, the functional design of the packaging can indirectly influence consumers’ judgment of product quality—the tightness of vacuum packaging can imply product freshness, while the rationality of compartmentalized packaging can reflect the professionalism of product pairings. These functional details, by enhancing consumers’ usage expectations and perceived experience, directly and positively impact purchase intent.

Based on the aforementioned multidimensional influence mechanisms, the environmental, functional, perceptual, and informational factors of packaging all operate through different pathways to affect consumers’ decision-making processes. Therefore, this paper proposes the following hypotheses:

H5a: Environmental factors of packaging have a positive impact on consumers’ behavioral intentions to purchase PRF foods.

H5b: Functional factors of packaging have a positive impact on consumers’ behavioral intentions to purchase PRF foods.

H5c: Perceptual factors of packaging have a positive impact on consumers’ behavioral intentions to purchase PRF foods.

H5d: Information factors of packaging have a positive impact on consumers’ behavioral intentions to purchase PRF foods.

#### Impact of external packaging factors on consumers’ healthy eating behaviors.

Consumer health-conscious eating behaviors are intrinsically a “cognitively driven-behaviorally transformed” process. Within this framework, packaging factors for ready-to-eat (RTE) foods emerge as critical intervention variables by shaping health perceptions, guiding choice preferences, and lowering behavioral execution thresholds. Their mechanisms of action align closely with the core tenets of healthy eating.

The positive impact of environmentally friendly packaging attributes on healthy eating behaviors stems from the cognitive association between eco-friendliness and health, as well as behavioral consistency effects. Existing research indicates a prevalent consumer psychological linkage where “eco-friendly equals healthy.” This implies that products utilizing environmentally conscious packaging are perceived as more likely to adhere to natural and sustainable production principles, leading to an inference that they meet higher health standards in raw material selection and processing [64]. For PRF, the “green” signal conveyed by eco-friendly packaging resonates with the core healthy eating aspiration of “minimal intervention and low additives.” This cognitive linkage steers consumers toward favoring such products, thereby reinforcing healthy dietary choice patterns. Furthermore, the usage of eco-friendly packaging (e.g., natural degradation of biodegradable materials, sorted disposal of recyclable packaging) cultivates a sense of “responsible consumption” among consumers. This consciousness extends to their eating habits, prompting greater attention to the dual impact of dietary choices on personal health and the environment, consequently reducing consumption of unhealthy foods high in oil, sugar, and excessive packaging waste, and promoting the formation of healthy eating behaviors.

The influence of functional factors on healthy eating behaviors centers on reducing the difficulty of healthy eating and enhancing behavioral sustainability through practical packaging design. Healthy eating often requires balancing “nutritional completeness” with “convenience and efficiency,” a need that functional packaging design for RTE foods can precisely address [65]. For instance, compartmentalized packaging enables the separate storage of ingredients (such as separating staples, vegetables, and proteins), ensuring reasonable nutritional balance while preventing flavor transfer and minimizing the time cost for consumers preparing healthy meals. Portion-controlled packaging assists consumers in managing intake, preventing overconsumption, and aligning with the “moderation principle” of healthy eating, thereby increasing consumers’ commitment to maintaining healthy dietary habits. The functional design of packaging directly dictates the convenience and experience of healthy eating, consequently influencing whether consumers incorporate it into their daily dietary choices.

Perceptual factors indirectly guide healthy eating behaviors by influencing consumers’ subjective judgments of the healthfulness of RTE foods. The visual characteristics of packaging exteriors shape consumer perceptions of a product’s health attributes through “synesthesia,” a mechanism particularly evident in RTE food consumption [66]. For example, cool color palettes like blue and green can evoke cognitive associations with “freshness, naturalness, and low calories,” whereas warm colors such as red and yellow are more readily linked with “high calories and high sugar content.” This difference in color perception directly impacts consumers’ assessments of the health level of RTE foods, consequently influencing dietary choices – consumers are more inclined to select products with cool-toned packaging to uphold healthy eating principles. Moreover, the perceived texture of packaging also affects health cognition: matte finishes and minimalist designs are often considered “more natural and less processed,” while overly ornate or reflective packaging materials may trigger negative associations of “excessive additives and unhealthiness.” This perceptual difference is further translated into preferential choices in eating behaviors, promoting the formation of healthy eating habits.

The guiding role of informational factors in healthy eating behavior stems from the core logic of “information empowering decision-making.” This means that health-related information on packaging enhances consumers’ health awareness, subsequently promoting the optimization of their dietary habits [64]. Health prompts on PRF food packaging (e.g., “Daily Recommended Intake,” “High Dietary Fiber”) help consumers quickly identify the health value of a product. For ordinary consumers, particularly those lacking specialized nutritional knowledge, such information lowers the cognitive threshold for healthy eating. The standardized presentation of nutrition labels (e.g., visualization of nutrient proportions) allows consumers to directly compare the healthiness of different PRF products, thereby enabling them to choose products that better align with their health needs. Furthermore, information labeling tailored to specific health goals (e.g., “Fat-Loss Friendly,” “Sugar-Control Formula”) can precisely match the health demands of specific demographic segments, guiding consumers to form targeted dietary selection habits. Existing research confirms that clear and accurate packaging health information significantly increases consumers’ probability of choosing healthy foods and reduces their intake of unhealthy foods. This conclusion has been fully validated in areas such as PRF ready-to-eat foods and meal replacement products.

Based on the analysis above, the environmental, functional, perceptual, and informational factors of packaging all influence consumers’ health eating cognition and behavioral choices. Therefore, this paper proposes the following hypotheses:

H6a: Environmental factors of packaging have a positive impact on consumers’ healthy eating behaviors.

H6b: Functional factors of packaging have a positive impact on consumers’ healthy eating behaviors.

H6c: Perceptual factors of packaging have a positive impact on consumers’ healthy eating behaviors.

H6d: Information factors of packaging have a positive impact on consumers’ healthy eating behaviors.

## Theoretical model

**Fig 1:**
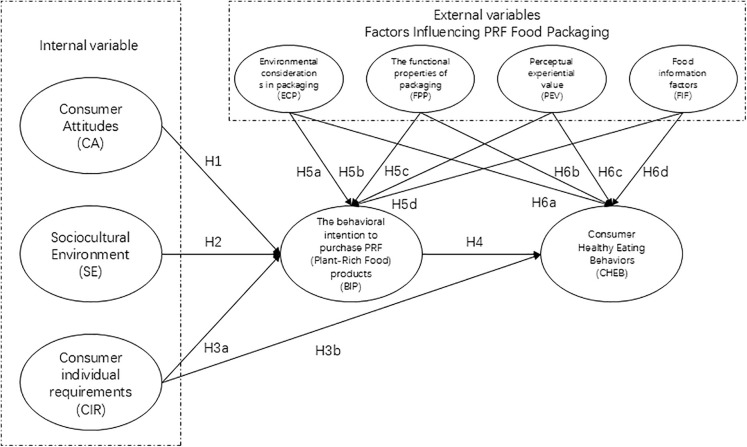
[href:javascript:;]Structural equation model.

Based on the analysis above, we construct the following theoretical model, as shown in Fig 1.

### Research method and questionnaire design

#### Research design.

This study adopted a quantitative cross-sectional survey design to examine the influence of plant-based food packaging attributes and consumer internal/external variables on purchase intention and healthy dietary behavior. Given the dispersed nature of plant-based food consumers, to obtain authentic and broad primary data, a convenience sampling method under non-probability sampling was employed with “voluntary participation and online accessibility” as core screening criteria. Questionnaires were disseminated through the open distribution function of the Wenjuanxing platform to potential PRF food consumers from different regions and consumption backgrounds. This sampling technique allows for rapid coverage of a wide target population and reduces research costs, while leveraging the traffic advantages of online platforms to compensate for the “difficult to reach and low efficiency” issues of probability sampling in dispersed group surveys, ensuring a balance between sample size and diversity.

#### Data collection.

A total of 594 questionnaires were recovered. Data screening criteria included: (1) logical inconsistencies or random responses (e.g., continuous selection of the same option); (2) completion time too short (below the pre-set reasonable completion time of 3–5 minutes); (3) duplicate submissions from the same IP address (only the first submission was retained). A final count of 566 valid questionnaires was obtained, resulting in a valid recovery rate of 95.3%. The sample size met Jackson’s standard of a parameter-to-sample ratio greater than 1:10 (566:27 ≈ 21:1), which is suitable for subsequent Structural Equation Model analysis.

#### Data analysis techniques.

This study employed a two-stage analysis approach. In the first stage, SPSS 24.0 software was utilized for descriptive statistical analysis, reliability analysis (Cronbach’s α), exploratory factor analysis (KMO-Bartlett test, variance extraction rate), and multicollinearity diagnosis (VIF). In the second stage, Partial Least Squares Structural Equation Modeling software, Smart-PLS 4.0, was used for confirmatory factor analysis (examining convergent and discriminant validity) and path hypothesis testing. Model fit was assessed using indicators such as SRMR, d-ULS, d-G, and NFI. This methodology is widely applied in the field of consumer behavioral intention research [67]. Hypothesis testing was conducted using the Bootstrap resampling method, with 5000 repetitions, and P < 0.05 was used as the standard for statistical significance.

#### The correlation of core psychological and behavioral variable scales.

The Consumer Attitude towards Plant-Based Foods (PRF) Scale (CA1-CA3) in this study is adapted from the relevant research of Barbosa [43], Consavage Stanley [9], and Czudec [44]. All three studies primarily focus on measuring attitudes within the domain of plant-based food consumption, aligning closely with the construct definition in our research. Barbosa’s [43] research centers on the association between consumers’ perception of the health value of plant-based foods and their attitudes, with scale items encompassing key dimensions such as “evaluation of health benefits” and “recognition of nutritional content.” This directly corresponds to our study’s definition of “attitude as a comprehensive evaluation of the health value and sensory experience of PRF.” Consavage Stanley’s [9] work emphasizes core factors influencing attitude formation in practical consumption scenarios, including taste, quality, and price. Their item design is tailored to consumers’ actual considerations during purchasing decisions, adding a dimension of practicality to attitude measurement. Czudec’s [44] research, on the other hand, focuses on the impact of environmental consciousness on attitudes towards plant-based foods, with scale items such as “acknowledgment of eco-friendly attributes,” fitting our study’s “environmental value dimension” of attitude. Collectively, these three studies comprehensively cover the key components of PRF attitudes from the perspectives of health, practicality, and environmental consciousness, providing a multi-dimensional theoretical basis for our scale items.

The Social Environment Scale (SE1-SE3) is adapted from studies by Karimah [47] and Muhammad [46] on social influence, which deeply align with our study’s construct definition of “social environment transmitting social pressure through channels such as family and reference groups.” Karimah’s [47] research systematically analyzes the impact of social relationships, such as family support and peer recommendations, on food consumption behavior. Its scale items explicitly cover specific contexts like “family dietary modeling” and “friends’ purchasing advice,” directly corresponding to the core influence pathways of the social environment in our study. Muhammad’s [46] research focuses on the role of social norms and group identity in consumption decisions, with items including measurement dimensions such as “social consensus” and “group behavior consistency,” supplementing the “social desirability pressure” measurement dimension within the social environment. These two studies, from the perspectives of “interpersonal influence” and “group norms,” respectively, fully encompass the core connotations of the social environment construct in our research. Their scale designs have undergone empirical validation and possess good reliability and validity, providing a reliable basis for precise measurement of the social environment.

The Consumer Intrinsic Needs Scale (CIR1-CIR3) is adapted from the research of Albornoz [51], Aysel Erciş [52], Anh [53], and Wang [54], and it highly matches our study’s construct definition of “personal needs encompassing multidimensional aspects such as health, environmental, price, and information.” Albornoz’s [51] research focuses on the driving role of health needs in plant-based food consumption, with scale items centered around “demand for health improvement” and “need for nutritional supplementation,” aligning with the core health needs dimension in our study. Aysel Erciş’s [52] research specifically explores consumption needs driven by environmental consciousness, with items including “practice of sustainable lifestyle” and “concern for environmental impact,” precisely corresponding to the environmental needs dimension. Anh’s [53] research concentrates on the relationship between price perception and consumption needs, with items involving “evaluation of cost-effectiveness” and “judgment of price reasonableness,” supplementing the measurement of price needs. Wang’s [54] research, conversely, examines the impact of information transparency on consumption decisions, with items such as “demand for product information access” and “clarity of ingredient labeling requirements,” completing the measurement content for information needs. From the core dimensions of health, environment, price, and information, these four studies comprehensively cover the multidimensional construct of personal needs in our research, ensuring that the scale can systematically capture consumers’ core needs and demands regarding PRF.

The Purchase Intention Scale (BIP1-BIP3), adapted from research by Park [45] and Wang [54] on plant-based food purchase intention, aligns perfectly with our study’s construct definition: “Purchase intention is the psychological inclination of consumers to willingly choose and prepare to purchase PRF.” Park's [45] study, grounded in the Theory of Planned Behavior, developed a measurement scale for plant-based food purchase intention, incorporating core dimensions such as “likelihood of future purchase” and “propensity for active choice,” which directly reflect the essence of purchase intention. Wang's [54] research, by integrating food packaging information features, added items concerning “purchase intention based on product attributes,” such as “willingness to purchase due to information transparency.” This is highly compatible with our study, which simultaneously considers both the PRF product itself and its packaging. Both studies focused on the plant-based food sector, and their validated scales exhibit strong adaptability. The item designs not only conform to the general measurement logic of purchase intention but also closely fit the specific context of PRF consumption, providing robust support for the precise measurement of purchase intention.

The Healthy Eating Behavior Scale (CHEB1-CHEB3), adapted from relevant studies by Dinu [58] and Satija [59], is deeply congruent with our study's construct definition: “Healthy eating behavior is a continuous dietary choice centered around PRF.” Dinu's [58] research, through systematic reviews and meta-analyses, established the association between plant-based dietary patterns and health outcomes. Its scale items revolve around behavioral indicators such as “frequency of daily PRF intake” and “proportion of PRF in dietary structure,” directly corresponding to the core measurement dimensions of healthy eating behavior in our study. Satija's [59] research focused on constructing a healthy plant-based eating index, with items including “regular consumption of whole grains, vegetables, and other PRFs” and “alternative behaviors to unhealthy eating,” supplementing the continuous and substitutive dimensions of healthy eating behavior. Both studies centered on plant-based eating behavior, and their scale designs, based on extensive empirical data, effectively capture the key characteristics of healthy eating behavior.

#### Source relevance of the external packaging factors scale.

The Environmental Concern Scale (ECP1-ECP3), adapted from research by Sparsh Rathi [62], aligns highly with our study's construct definition: “Packaging environmental factors refer to the sustainable attributes of packaging materials and design.” Sparsh Rathi's [62] research specifically investigated the impact of the environmental characteristics of food packaging on consumer decisions. Its scale items cover core dimensions such as “recognition of degradable materials,” “attention to eco-labels,” and “perception of carbon footprint,” precisely corresponding to the core aspects of PRF packaging environmental factors in our study. Through empirical validation, this research confirmed that these items can effectively measure consumers’ perceptions and preferences for the environmental attributes of packaging. This aligns perfectly with our study's objective of exploring the influence of packaging environmental factors on purchase intention and behavior, providing direct theoretical and empirical grounds for measuring environmental factors.

The Functional Factors Scale (FPP1-FPP3) is adapted from the research by Broucke [35], aligning deeply with our study's construct definition of “packaging functional factors referring to the core functions of packaging such as protection, convenience, and utility.” Broucke's [35] research systematically analyzed the functional characteristics of food packaging. Its scale items encompass key dimensions like “barrier properties (oxidation and moisture resistance),” “ease of opening and re-sealing,” and “storage convenience,” fully covering the core content of packaging functional factors in our study. The research points out that the core functions of packaging directly impact consumer experience and purchasing decisions. Its item design closely matches the actual usage scenarios of food consumption, making it highly compatible with the PRF's requirement for packaging to simultaneously protect food quality and enhance ease of use.

The Perceptual Factors Scale (PEV1-PEV3) is adapted from the research by Azad [61] and Chu [66], closely matching our study's construct definition of “packaging perceptual factors referring to the overall evaluation of packaging formed by consumers through sensory experience.” Azad's [61] research focuses on the visual and tactile perception of packaging, with items including “color scheme attractiveness” and “material texture comfort,” corresponding to the visual and tactile stimulation dimensions in sensory marketing theory. Chu's [66] research, on the other hand, concerns the overall texture and emotional associations of packaging, with items covering “quality perception” and “natural association,” supplementing the emotional and value-related dimensions of perceptual factors. Together, from the perspectives of “sensory experience” and “emotional association,” these scales comprehensively cover the core content of packaging perceptual factors in our study. Their item design aligns with the core logic of embodied cognition theory, effectively capturing the impact of packaging sensory stimuli on consumer cognition and emotion, and is highly compatible with our study's integrated multi-theoretical framework.

The Informational Factors Scale (FIF1-FIF3) is adapted from the research by Sparsh Rathi [62], perfectly aligning with our study's construct definition of “packaging informational factors referring to product-related information conveyed by packaging.” Sparsh Rathi's [62] research includes measurement items for the effectiveness of information delivery in food packaging, covering core dimensions such as “clarity of nutritional ingredient presentation,” “explicitness of health claim statements,” and “practicality of usage instructions.” These precisely correspond to the core content of packaging informational factors in our study. Based on information processing theory, this research confirms that clear and relevant packaging information can activate consumers’ central route processing, which is consistent with our study's logic of exploring how packaging information influences purchasing decisions through cognitive processing. Its item design can effectively measure key aspects of information processing, providing a solid foundation for the scientific measurement of informational factors (Table 1).

**Table 1:** Definition of variable operability and reference scales.

Construct	items	Source
Consumer Attitudes (CA)	CA1: I think foods rich in plants are a healthy choice. CA2: I have a positive view of foods rich in plants. CA3: I believe consuming foods rich in plants is beneficial to the environment.	[9,43,44]
Sociocultural Environment (SE)	SE1: The opinions of the people around me regarding plant-based foods will influence my perception. SE2: The dietary habits of family members and friends will affect my choice of plant-based foods. SE3: The publicity and promotion of plant-based foods in society will influence my purchasing decisions.	[46,47]
Consumer individual requirements (CIR)	CIR1: I choose plant-rich food because it can meet my nutritional needs. CIR2: Consuming plant-rich food is in line with my lifestyle. CIR3: I believe plant-rich food can fulfill my pursuit of a healthy diet.	[51–54]
The behavioral intention to purchase PRF (Plant-Rich Food) products (BIP)	BIP1: I have the intention to purchase foods rich in plants. BIP2: In the future, I might increase the frequency of purchasing foods rich in plants. BIP3: I will recommend to others to purchase foods rich in plants.	[45,54]
Consumer Healthy Eating Behaviors (CHEB)	CHEB1: I will actively choose healthy foods, including those rich in plants. CHEB2: I will pay attention to my diet structure and ensure adequate intake of nutrients. Plant-rich foods are part of it. CHEB3: I will try to avoid unhealthy foods and prefer healthier foods like those rich in plants.	[58,59]
Environmental considerations in packaging (ECP)	ECP1: Whether the packaging materials of plant-rich food are environmentally friendly will affect my purchasing decision. ECP2: I prefer to buy plant-rich food with recyclable packaging. ECP3: The environmental friendliness of the packaging is an important consideration for me when choosing plant-rich food.	[62]
The functional properties of packaging (FPP)	FPP1: The portability of the packaging for plant-rich food will affect my purchase decision. FPP2: Plant-rich food with good preservation function in the packaging is more likely to attract me to buy it. FPP3: Whether the packaging is easy to open is a factor I consider when choosing plant-rich food.	[35]
Perceptual experiential value (PEV)	PEV1: The attractive packaging design of plant-rich food will increase my desire to purchase it. PEV2: The good texture of the packaging will make me believe that the quality of the plant-rich food is higher, thus making me more willing to buy it. PEV3: My first impression of the packaging of plant-rich food will influence my purchasing decision.	[61,66]
Food information factors (FIF)	FIF1: The clear nutritional information on the packaging will help me make a decision to purchase plant-rich foods. FIF2: The information such as the consumption method on the packaging is very important for my purchase of plant-rich foods. FIF3: I will be more willing to purchase plant-rich foods because of the complete and accurate information on the packaging.	[62]

#### Informed consent.

The study was approved by the Faculty of Art & Ceramic design, Jingdezhen University with ID:No.JDZU-E-2024–1021 and Oct 10, 2024. The research was conducted from February to May 2025.

Given the dispersed distribution of individuals consuming and purchasing PRF foods, a comprehensive and authentic collection of consumer feedback, as well as the acquisition of accurate primary data, was deemed necessary. Following thorough deliberation and analysis, this study adopted an online questionnaire survey methodology to conduct consumer research centered on PRF foods and their packaging. The research was implemented between February and May 2025. Questionnaires were widely distributed to the general public via the Wenjuanxing online questionnaire platform. This approach served a dual purpose: firstly, to surmount geographical limitations, thereby ensuring a broad spectrum of sample origins and guaranteeing the comprehensiveness of data coverage; and secondly, to enhance the reliability and representativeness of the research findings through the natural selection afforded by a large sample size. To safeguard participants’ right to information and autonomous choice, an online informed consent module was incorporated at the commencement of the questionnaire. This module elucidated key information, including the research objectives, scope of inquiry, data utilization (solely for academic research, with anonymization), and participant rights (including the right to withdraw at any time, with data not retained upon withdrawal). Considering the specific nature of online research, this study employed an online check-box confirmation mechanism. Upon reviewing the informed consent statement, participants were required to explicitly and fully comprehend all information and to select the “Agree to Participate” option to proceed to the questionnaire; selecting “Decline to Participate” automatically terminated the survey without recording any personal information. This procedure strictly adheres to the principle of “complete voluntariness,” ensuring that participants autonomously decide whether to participate based on full information, free from any coercive or suggestive actions.

## Research results

### Sample demographic characteristics

This study analyzed 566 valid questionnaires using SPSS 24.0, beginning with a descriptive analysis to ascertain the demographic characteristics of the survey sample. The questionnaire comprised 27 scale questions from the 566 valid samples collected, meeting Jackson's criterion of a parameter-to-sample size ratio exceeding 1:10 [68]. Consequently, the subsequent data analysis is based on this foundation, as illustrated in Table 2, which presents the descriptive statistical analysis of the sample's demographic variables.

**Table 2:** Demographic profile of sample（n = 566）.

Sample	Category	Number	Percentage%
Gender	Male	318	56.2
	Female	248	43.8
Age	18-24	101	17.8
	25-34	114	20.1
	35-44	166	29.3
	45-54	116	20.5
	Over 55	69	12.2
Occupation	Student	71	12.5
	Freelance or self-employed	108	19.1
	Public officials or public institutions	95	16.8
	Multinational enterprises and private enterprises	242	42.8
	Others	50	8.8
Education	High school and below	132	23.3
	Associate degree	142	25.1
	Undergraduate degree	195	34.5
	Master's degree candidate	72	12.7
	Doctoral degree or above	25	4.4
Frequency of consuming PRF food	Consumed every day	140	24.7
	Consumed three times a week	213	37.6
	Consumed once a week	60	10.6
	Consumed once a month	96	17.0
	Consumed once or twice every half a year	57	10.1
Time of exposure to PRF food	Within three months	61	10.8
	Three to six months	80	14.1
	Six to twelve months	46	8.1
	One to two years	49	8.7
	More than two years	198	35.0
	More than five years	132	23.3

Overall, the distribution of the survey sample across key dimensions such as gender, age, occupation, education level, and consumption behavior aligns remarkably well with the target consumer profile for PRF foods and general patterns within the food market. The validity and representativeness of the sample are primarily evident in three aspects: Firstly, the sample size is adequate and appropriate (566 individuals), meeting the fundamental requirements for empirical research. Furthermore, the sample distribution across core dimensions is relatively balanced, avoiding extreme skewness and effectively minimizing sampling error. Secondly, the sample encompasses both the core consumer group for PRF foods (aged 25–54, employed professionals, individuals with mid-to-high education levels, and frequent long-term consumers) and potential consumer segments (younger demographics, students, and infrequent short-term users). This dual focus ensures that the research findings are applicable to diverse types of PRF consumers. Thirdly, the consumption behavior characteristics (high-frequency stable, low-frequency dispersed) and contact time distribution within the sample are consistent with the current market penetration status of PRF foods. This accurately reflects consumer acceptance and usage habits, providing a robust data foundation for subsequent theoretical model validation and practical strategy development.

Based on the survey results of PRF food consumers, the following observations can be made: Among the 566 respondents, males slightly outnumbered females, with 318 males (56.2%) and 248 females (43.8%), indicating a moderate gender disparity. Analyzing the age structure, the survey population primarily comprised mature individuals aged 25–54, with the 35–44 age group as the core, accounting for 166 individuals (29.3%), the highest among all age groups. The 25–34 (114 individuals, 20.1%) and 45–54 (116 individuals, 20.5%) age groups had similar proportions, with these three groups collectively representing nearly 70%. The younger (18–24, 101 individuals, 17.8%) and older (55 + , 69 individuals, 12.2%) age groups had relatively lower proportions, aligning with the typical characteristics of food consumer demographics. Regarding occupation, employees from multinational and private enterprises led significantly, with 242 individuals (42.8%), forming the core consumer group for PRF food. Freelancers or self-employed individuals followed, accounting for 108 individuals (19.1%). Public sector employees or those in public institutions (95 individuals, 16.8%) and students (71 individuals, 12.5%) followed in descending order. Other occupations had the lowest proportion, at only 8.8% (50 individuals), indicating a good match between occupational distribution and the consumption capacity and needs of different groups. In terms of education, the survey population exhibited a clear trend of “high and medium education dominance”: over 50% held a bachelor's degree or higher, with the highest number of individuals holding a bachelor's degree (195 individuals, 34.5%), followed by master's (72 individuals, 12.7%) and doctoral degrees or higher (25 individuals, 4.4%). Those with associate degrees accounted for 25.1% (142 individuals), while those with a high school education or below accounted for 23.3% (132 individuals), reflecting the varying acceptance of PRF food among different educational levels. Regarding consumption frequency, PRF food users primarily consumed it frequently: the group consuming it three times a week had the highest proportion, at 37.6% (213 individuals); those consuming it daily accounted for 24.7% (140 individuals), with these two high-frequency consumption groups accounting for over 60% combined, indicating that most users have developed a stable consumption habit. Low-frequency consumption groups were relatively dispersed, with those consuming it once a month accounting for 17.0% (96 individuals), and those consuming it once a week (60 individuals, 10.6%) and once or twice every six months (57 individuals, 10.1%) having even lower proportions, which is consistent with the general pattern of “high frequency is stable, low frequency is dispersed” in food consumption. In terms of exposure time, the group with long-term exposure to PRF food had a clear advantage: respondents with over two years of exposure reached 198 individuals, accounting for 35.0%; those with over five years of experience numbered 132, accounting for 23.3%, with the two groups combined accounting for nearly 60%, demonstrating PRF food's strong appeal to long-term users. The proportion of short-term exposure groups was relatively balanced, with those exposed for three to six months accounting for 14.1% (80 individuals), and those exposed for less than three months (61 individuals, 10.8%), one to two years (49 individuals, 8.7%), and six to twelve months (46 individuals, 8.1%) decreasing in proportion, reflecting that a certain number of new users are still in the trial phase.

Considering the consumption patterns of food in real-world society, groups that frequently consume specific foods often develop stable consumption habits over time. The proportion of infrequent consumption and short-term exposure also highly aligns with the trial consumption characteristics of new consumer groups. Therefore, the sample distribution across the dimensions of this questionnaire survey is reasonable, and the data is highly representative, fully meeting the needs of subsequent research. Based on the current population structure, consumption frequency, and exposure time survey results, it can be determined that conducting this research on PRF foods has significant practical implications and necessity.

### Reliability and validity analysis

Reliability reflects the consistency of results obtained by a measurement tool during repeated use, encompassing the accuracy, stability, and robustness of the measurement results [69]. To verify the reliability of the scales in this study, the researchers used SPSS 22.0 software to calculate the Cronbach's α values for each measurement variable. Existing research indicates that when the Cronbach's α value is greater than 0.6, the scale data can be determined to have accuracy and validity [70]. As can be seen from the survey data in Table 3, the Cronbach's α values for all measurement variables are above 0.7; at the same time, the “total correlation after item deletion” for each item is greater than 0.5, and the overall Cronbach's α value of the scale does not exceed the current value after deleting any item. These two results indicate that the items in the questionnaire do not need to be deleted, further proving that the scales used in this study have good reliability. Simultaneously, the Variance Inflation Factor (VIF) is an important indicator for assessing multicollinearity issues among independent variables, which can be used to measure the extent to which each predictor variable is linearly explained by other predictor variables. The higher the VIF value, the more severe the multicollinearity problem [71]. Related research suggests that when the VIF value exceeds 5, it indicates that there is a certain degree of multicollinearity within the measurement scale [72]; while ideally, the VIF value should be below 3.3 [73], at which point the correlation between predictor variables is low, and the regression estimation results are more stable, which helps to build a robust analysis model. Similarly, combining the data in Table 3, it can be seen that the VIF values of all variables are below 2.638, indicating that there is no multicollinearity problem in the scale. In summary, from the test results of Cronbach's α value and VIF value, it can be concluded that the measurement scales used in this study have good reliability and are free from multicollinearity interference, providing strong support for constructing a stable and reliable analysis model.

**Table 3:** Reliability analysis results（n = 566）.

Dimension	Items	Collinearity Statistics (VIF)	Corrected Item-to-Total Correlation	Cronbach’s α if Item Deleted	Cronbach’s α
CA	CA1	2.352	0.757	0.844	0.881
	CA2	2.437	0.766	0.836	
	CA3	2.638	0.788	0.816	
SE	SE1	2.325	0.754	0.807	0.868
	SE2	2.318	0.753	0.808	
	SE3	2.167	0.734	0.826	
CIR	CIR1	2.328	0.755	0.812	0.869
	CIR2	2.271	0.748	0.818	
	CIR3	2.266	0.747	0.819	
BIP	BIP1	2.357	0.759	0.793	0.864
	BIP2	2.152	0.731	0.819	
	BIP3	2.185	0.735	0.815	
CHEB	CHEB1	2.142	0.730	0.795	0.855
	CHEB2	2.099	0.724	0.800	
	CHEB3	2.127	0.728	0.796	
ECP	ECP1	2.128	0.726	0.816	0.862
	ECP2	2.371	0.760	0.784	
	ECP3	2.128	0.726	0.816	
FPP	FPP1	2.291	0.750	0.816	0.869
	FPP2	2.232	0.743	0.823	
	FPP3	2.346	0.757	0.809	
PEV	PEV1	2.181	0.734	0.827	0.868
	PEV2	2.478	0.772	0.792	
	PEV3	2.217	0.738	0.823	
FIF	FIF1	2.209	0.740	0.801	0.860
	FIF2	2.167	0.734	0.806	
	FIF3	2.168	0.734	0.806	

Exploratory factor analysis was conducted using SPSS 22.0, employing the Kaiser-Meyer-Olkin (KMO) and Bartlett's test of sphericity to ascertain the suitability of the data for factor extraction [74]. Prior research suggests that a KMO value exceeding 0.5, coupled with a Bartlett's test of sphericity significance level less than 0.05 and not approaching zero, indicates that the data is appropriate for factor analysis [75,76]. Furthermore, when each variable extracts only one factor with an eigenvalue greater than 1, and the cumulative variance contribution rate of each variable exceeds 50%, it suggests that the extracted factors effectively explain the variables. Additionally, if the communality of all items is greater than 0.5 and the factor loading is greater than 0.6, this indicates good unidimensionality [77]. As shown in Table 4, the KMO values for the variables ranged from 0.734 to 0.743, all exceeding the 0.5 threshold. The Bartlett's test of sphericity yielded significance levels less than 0.05 and not approaching zero, with all variables showing significant results, indicating a solid foundation for factor analysis. Subsequently, principal component analysis was employed for factor analysis of each variable. The results revealed that each variable extracted only one factor with an eigenvalue greater than 1, and the cumulative variance contribution rate for each variable was at least 77.539%, exceeding 50%. This suggests that the factors extracted from the two scales in this study effectively explain the variables. The communality of all items was at least 0.772, exceeding 0.5, and the factor loading was at least 0.878, exceeding 0.6, all within the recommended range. Therefore, the survey results of this study demonstrate good unidimensionality.

**Table 4:** Exploratory factor analysis result（n = 566）.

Dimension	Items	KMO	Bartlett Sphere Test	Factor Loading	Commonality	Eigenvalue	Total variation explained％
CA	CA1	0.743	0	0.892	0.796	2.426	80.861
	CA2			0.897	0.805		
	CA3			0.909	0.826		
SE	SE1	0.739	0	0.893	0.798	2.372	79.063
	SE2			0.893	0.797		
	SE3			0.882	0.777		
CIR	CIR1	0.740	0	0.893	0.798	2.379	79.300
	CIR2			0.889	0.791		
	CIR3			0.889	0.790		
BIP	BIP1	0.736	0	0.896	0.803	2.359	78.626
	BIP2			0.881	0.775		
	BIP3			0.883	0.780		
CHEB	CHEB1	0.734	0	0.882	0.778	2.326	77.539
	CHEB2			0.878	0.772		
	CHEB3			0.881	0.776		
ECP	ECP1	0.734	0	0.878	0.772	2.349	78.314
	ECP2			0.898	0.806		
	ECP3			0.878	0.772		
FPP	FPP1	0.740	0	0.891	0.793	2.379	79.298
	FPP2			0.886	0.786		
	FPP3			0.895	0.800		
PEV	PEV1	0.736	0	0.881	0.777	2.374	79.129
	PEV2			0.903	0.815		
	PEV3			0.884	0.782		
FIF	FIF1	0.737	0	0.887	0.786	2.346	78.203
	FIF2			0.883	0.780		
	FIF3			0.883	0.780		

Employing confirmatory factor analysis, the factor loadings of each item in the scale were extracted. All factor loadings exceeded 0.5, indicating that all items within each variable consistently explain the variable. This consistency not only demonstrates that the items effectively reflect the essence of the variable but also suggests that the scale possesses high stability and reliability in measuring the variable [78]. Relevant research indicates that composite reliability (CR) and average variance extracted (AVE) are crucial metrics for assessing whether related variables exhibit good convergent validity. The CR value should not be less than 0.7, and the AVE value should not be below 0.5 [79,80]. Therefore, in conjunction with the results presented in Table 5, calculations were performed based on the factor loadings of each item, yielding the composite reliability (CR) and average variance extracted (AVE). The factor loadings in this study all exceeded 0.5, and the CR values were all greater than 0.778, while the square root of AVE values all surpassed 0.558, exceeding the thresholds of CR (0.7) and AVE (0.5). This indicates that the related variables demonstrate robust convergent validity.

**Table 5:** Convergent validity analysis results (n = 566).

Dimension	Items	UnstandardizedFactor Loading	Standardize Factor Loading	*SE*	*P-Value*	AVE	CR
CA	CA1	1	0.778	–	–		
	CA2	1.038	0.789	0.051	0	0.624	0.833
	CA3	1.019	0.803	0.049	0		
SE	SE1	1	0.747	–	–	0.564	0.795
	SE2	1.037	0.765	0.054	0		
	SE3	0.986	0.740	0.054	0		
CIR	CIR1	1	0.758	–	–	0.560	0.793
	CIR2	0.987	0.761	0.051	0		
	CIR3	0.958	0.726	0.052	0		
BIP	BIP1	1	0.770	–	–	0.561	0.793
	BIP2	0.964	0.739	0.051	0		
	BIP3	0.942	0.738	0.049	0		
CHEB	CHEB1	1	0.741	–	–	0.541	0.780
	CHEB2	0.931	0.733	0.051	0		
	CHEB3	0.932	0.733	0.051	0		
ECP	ECP1	1	0.822	–	–	0.675	0.862
	ECP2	1.040	0.835	0.046	0		
	ECP3	0.975	0.808	0.045	0		
FPP	FPP1	1	0.740	–	–	0.558	0.791
	FPP2	0.969	0.736	0.054	0		
	FPP3	0.989	0.765	0.053	0		
PEV	PEV1	1	0.826	–	–	0.688	0.869
	PEV2	1.039	0.843	0.042	0		
	PEV3	0.995	0.819	0.042	0		
FIF	FIF1	1	0.832	–	–	0.683	0.861
	FIF2	0.930	0.810	0.039	0		
	FIF3	0.958	0.819	0.040	0		

0.5 is the lowest standard for AVE and CR value > 0.7.

As indicated in Table 6, the outer loadings derived from the respective indicators within each construct in this study surpassed the cross-loadings with every additional construct. It is noteworthy that cross-loadings are frequently utilized as an initial procedural step in the rigorous evaluation of the discriminant validity of indicators, a methodology supported by prior research [81]. Table 6 specifically details these discriminant validity assessments, focusing on cross-loadings.

**Table 6:** Discriminant validity: Cross loading（N = 566）.

	BIP	CA	CHEB	CIR	ECP	FIF	FPP	PEV	SE
BIP1	0.897	0.783	0.781	0.784	0.786	0.783	0.793	0.783	0.784
BIP2	0.881	0.745	0.766	0.769	0.770	0.766	0.757	0.766	0.750
BIP3	0.882	0.756	0.760	0.764	0.757	0.765	0.771	0.757	0.753
CHEB1	0.758	0.763	0.883	0.761	0.777	0.774	0.767	0.761	0.769
CHEB2	0.761	0.766	0.878	0.748	0.760	0.774	0.747	0.752	0.758
CHEB3	0.773	0.745	0.881	0.762	0.760	0.763	0.749	0.768	0.759
ECP1	0.751	0.757	0.768	0.784	0.878	0.756	0.775	0.765	0.755
ECP2	0.788	0.770	0.780	0.791	0.898	0.801	0.788	0.790	0.779
ECP3	0.770	0.751	0.760	0.757	0.879	0.771	0.768	0.762	0.772
FPP1	0.780	0.752	0.763	0.758	0.775	0.757	0.891	0.770	0.763
FPP2	0.764	0.741	0.762	0.763	0.774	0.776	0.886	0.756	0.755
FPP3	0.786	0.778	0.765	0.793	0.796	0.792	0.895	0.778	0.771
PEV1	0.771	0.765	0.765	0.771	0.759	0.763	0.758	0.882	0.769
PEV2	0.788	0.791	0.777	0.782	0.802	0.786	0.776	0.903	0.784
PEV3	0.753	0.770	0.763	0.763	0.768	0.759	0.767	0.883	0.752
FIF1	0.780	0.775	0.779	0.776	0.781	0.888	0.777	0.780	0.772
FIF2	0.764	0.759	0.773	0.762	0.785	0.883	0.771	0.754	0.759
FIF3	0.764	0.766	0.769	0.772	0.760	0.883	0.760	0.761	0.757
CA1	0.751	0.890	0.769	0.768	0.762	0.764	0.762	0.789	0.767
CA2	0.776	0.898	0.772	0.762	0.773	0.771	0.751	0.785	0.788
CA3	0.789	0.910	0.781	0.780	0.780	0.803	0.779	0.778	0.778
SE1	0.766	0.778	0.765	0.766	0.768	0.768	0.751	0.761	0.893
SE2	0.778	0.761	0.787	0.787	0.794	0.780	0.785	0.775	0.894
SE3	0.751	0.767	0.756	0.779	0.756	0.752	0.750	0.769	0.880
CIR1	0.779	0.768	0.776	0.894	0.782	0.795	0.781	0.774	0.787
CIR2	0.787	0.767	0.777	0.891	0.795	0.777	0.786	0.786	0.785
CIR3	0.760	0.753	0.744	0.886	0.769	0.754	0.746	0.759	0.763

The Heterotrait-Monotrait Ratio (HTMT) serves as a crucial index for rigorously evaluating the discriminant validity of constructs integrated within a structural equation modeling (SEM) framework. Generally, HTMT values falling below a threshold of 0.85 are interpreted as evidence that the variables under consideration possess adequate discriminant validity [82]. This evaluation is paramount in mitigating the potential for multicollinearity, a phenomenon that can arise when constructs exhibit high intercorrelations, thereby compromising the integrity of the model. Our analysis reveals that all computed HTMT scores adhere to acceptable parameters, remaining at or below the 0.85 benchmark, with individual values spanning from 0.664 to 0.845 as detailed in Table 7. Consequently, we can confidently assert that the empirical evidence gathered in this investigation sufficiently substantiates the model's satisfactory discriminant validity.

**Table 7:** Discrminant validty-Heterotrait ratio（n = 566）.

Latent variable	BIP	CA	CHEB	CIR	ECP	FIF	FPP	PEV	SE
**BIP**									
**CA**	0.727								
**CHEB**	0.675	0.698							
**CIR**	0.707	0.694	0.672						
**ECP**	0.687	0.687	0.677	0.689					
**FIF**	0.694	0.721	0.708	0.726	0.674				
**FPP**	0.845	0.831	0.800	0.837	0.828	0.825			
**PEV**	0.692	0.697	0.664	0.693	0.665	0.683	0.824		
**SE**	0.695	0.676	0.673	0.701	0.648	0.681	0.804	0.665	

Based on the Fornell-Larcker criterion, discriminant validity assesses the degree of differentiation among latent variables. If the square root of the average variance extracted (AVE) for all latent variables is no greater than 0.9, the scale demonstrates good discriminant validity [80]. As shown in Table 8, the square root of the AVE for each latent variable is greater than the correlation coefficients between the corresponding latent variables. This indicates that the main variables have significant associations while maintaining sufficient differentiation, and all correlation coefficients do not exceed 0.9. This suggests that the research model can clearly define different constructs, effectively avoiding confusion between variables, and ensuring the scientific validity and rigor of the research measurement tools, thus providing a reliable basis for subsequent data analysis and theoretical validation.

**Table 8:** Correlation coefficient and average extraction variance（n = 566）.

Latent variable	BIP	CA	CHEB	CIP	ECP	FIF	FPP	PEV	SE
**BIP**	0.887								
**CA**	0.859	0.899							
**CHEB**	0.867	0.861	0.881						
**CIP**	0.871	0.856	0.860	0.890					
**ECP**	0.870	0.858	0.869	0.878	0.885				
**FIF**	0.870	0.867	0.875	0.871	0.877	0.884			
**FPP**	0.873	0.850	0.857	0.866	0.878	0.870	0.890		
**PEV**	0.867	0.872	0.864	0.868	0.873	0.865	0.862	0.890	
**SE**	0.860	0.865	0.865	0.874	0.869	0.862	0.857	0.864	0.889

### Model testing

Table 9 presents Q^2^, R^2^, and the Goodness of Fit (GOF). Q^2^ assesses the predictive relevance of endogenous constructs. R^2^ measures the extent to which all independent variables in the model explain the variance of the dependent variable. Relevant research indicates that an R^2^ value greater than 0.33 is considered good, and greater than 0.6 is ideal [83]. As shown in Table 9, BIP's R^2^ value is 0.849, indicating it explains 84.9% of the variance in outcomes, meeting the standard for an ideal explanation. CHEB's R^2^ value is 0.841, signifying it explains 84.1% of the variance in outcomes, also meeting the standard for an ideal explanation. Furthermore, a Q^2^ value greater than 0 indicates the model possesses predictive capability [83] The overall GOF = 0.682, exceeding the 0.36 benchmark for high fit [84], which suggests a robust model fit.

**Table 9:** Explanation of variance.

	R Square	R Square Adjusted	Q² Predict	GOF
The behavioral intention to purchase PRF (Plant-Rich Food) products **(BIP)**	0.849	0.847	0.845	0.682
Consumer Healthy Eating Behaviors **(CHEB)**	0.841	0.839	0.835	

R^2^ value less than 0.19 indicates a weak explanation; values between 0.33 and 0.6 represent a good explanation; and values greater than 0.6 indicate an ideal explanation.

Relevant research indicates that the Standardized Root Mean Square Residual (SRMR) serves as an index for assessing model fit. A lower SRMR value suggests a better fit of the model to the data; specifically, an SRMR value below 0.08 indicates a good fit [85,86]. A d-ULS value below 0.95 suggests a good model fit with an appropriate number of free parameters [87]. Similarly, a d-G value below 0.95 indicates a good model fit with a suitable number of free parameters [88]. Furthermore, a Normed Fit Index (NFI) greater than 0.8 indicates a high degree of model fit, with values closer to 1 signifying a stronger fit, implying that the model accurately captures the structure and relationships within the data [89]. Therefore, as shown in Table 10, the SRMR value of 0.031 is below the threshold value of 0.08, indicating that the model has a high degree of fit; the d-ULS value of 0.357 and the d-G value of 0.555 are both less than the threshold value of 0.95, indicating a good fit; the NFI value of 0.889 is greater than 0.8, indicating that the model has a good degree of fit.

**Table 10:** Model fit measures.

Common Indices	d-ULS	d-G	SRMR	NFI
Criteria	<0.95	<0.95	<0.08	>0.8
Values	0.357	0.555	0.031	0.889

### Path hypothesis analysis

**Fig 2:**
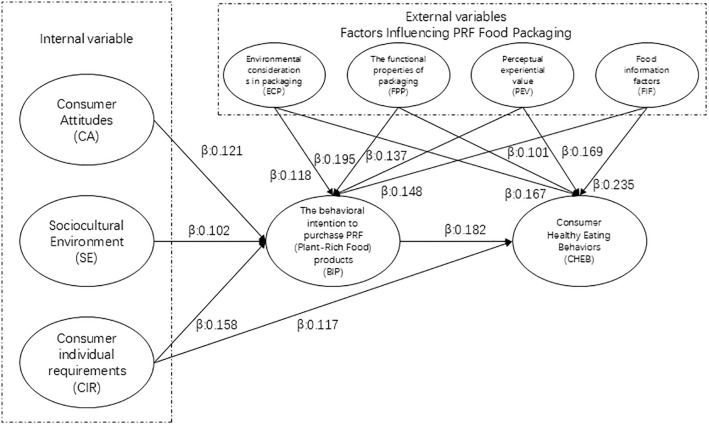
Hypothesis test results.

Finally, the PLS-SEM method was used to test the hypotheses and calculate the path coefficients. Using the Bootstrapp method, 5000 random samples were drawn independently. As shown in Table 11, all the path P-values in this study were less than 0.05, which is statistically significant. Therefore, all the hypotheses of this study were verified. (Fig 2)

**Table 11:** Hypothesis model path relationship test.

Hypothesis	Path	f²	β Co-Efficient	T statistics	*P values*	Decision
H1	CA → BIP	0.016	0.121	0.040	***	Accept
H2	SE → BIP	0.011	0.102	0.042	*,*0.014*	Accept
H3a	CIR → BIP	0.025	0.158	0.042	***	Accept
H3b	CIR→CHEB	0.013	0.117	0.043	**,*0.007*	Accept
H4	BIP→CHEB	0.033	0.182	0.043	***	Accept
H5a	ECP → BIP	0.013	0.118	0.042	**,*0.005*	Accept
H5b	FPP → BIP	0.040	0.195	0.038	***	Accept
H5c	PEV → BIP	0.019	0.137	0.043	***,*0.001*	Accept
H5d	FIF → BIP	0.022	0.148	0.042	***	Accept
H6a	ECP→CHEB	0.025	0.167	0.045	***	Accept
H6b	FPP→CHEB	0.010	0.101	0.042	*,*0.016*	Accept
H6c	PEV→CHEB	0.029	0.169	0.040	***	Accept
H6d	FIF→CHEB	0.054	0.235	0.044	***	Accept

Significance of P value: *** represents P≤0.001, ** represents P≤0.01, * represents P ≤ 0.05.

The results of hypothesis testing are shown in the above table, as shown in Fig 2.

## Discussion

The empirical findings from the PLS-SEM analysis confirm all hypotheses (H1-H6d), demonstrating positive influences among all variables. This validation substantiates the theoretical framework we constructed, centered on the Theory of Planned Behavior (TPB) and integrating Information Processing Theory with Sensory Marketing Theory, which posits a pathway from “Individual Needs – Product Attributes – Purchase Intention – Healthy Behavior.” The core discoveries highlight significant variations in the strength of influence across these pathways. Discussed below are the deeper implications of these findings, considered in conjunction with theoretical logic and practical applications:

(1)The impact of core variables on the purchase intention of plant-based foods (BIP) reveals a hierarchical structure of driving forces. The study, through path analysis, confirms that seven variables—Consumer Attitudes (CA), Sociocultural Environment (SE), Consumer Individual Requirements (CIR), Environmental Considerations in Packaging (ECP), Perceptual Experiential Value (PEV), Food Information Factors (FIF), and The Functional Properties of Packaging (FPP)—all significantly and positively influence BIP, yet the intensity and practical value of each variable exhibit a clear hierarchical pattern. From the perspective of hypothesis verification data, a clear gradient exists in the influence strength of positive driving factors. Among all variables, the driving effect of packaging functional attributes (FPP) is most prominent, exhibiting the highest path coefficient (β = 0.195) and T-statistic (T = 5.105) (P = 0.000). Its effect size, f^2^ = 0.040, although representing a small effect, is the largest among BIP driving variables, indicating that FPP possesses the relatively strongest unique explanatory power for BIP compared to other variables. This finding aligns closely with the practical characteristic of FPP directly linking to users’ core needs (e.g., freshness preservation, portability), validating the judgment of FPP's critical position in the preliminary theoretical hypotheses. Following closely are consumer individual needs (CIR) (β = 0.158, T = 3.762, P = 0.000) and food information factors (FIF) (β = 0.148, T = 3.553, P = 0.000). The path coefficients of these two are similar, with CIR's f^2^ = 0.025 and FIF's f^2^ = 0.022. Both are small effects and the gap in f^2^ from FPP is relatively small, suggesting that although there is a gradient in path coefficients among the three, the actual effect size differences are limited. This reflects that users’ functional needs for PRF (e.g., nutrition, taste) and product information transparency (e.g., ingredient labels, nutritional content) are important “need-based” and “decision-based” drivers that foster purchase intention. Perceived experiential value (PEV) (β = 0.137, T = 3.194, P = 0.001) and consumer attitudes (CA) (β = 0.121, T = 4.244, P = 0.000) exhibit moderate path coefficients. PEV's f^2^ = 0.019 and CA's f^2^ = 0.016 are both close to the small effect threshold of 0.02, with effect sizes slightly lower than CIR and FIF, indicating their unique contributions to BIP are limited. Among these, Consumer Attitudes (CA), as a foundational influence variable, embodies the “foundational supportive” role of users’ overall positive evaluation of PRF in purchase intention, while PEV highlights the supplementary driving value of subjective experiences such as taste and visual appeal in purchase decisions. Packaging consideration (ECP) (β = 0.118, T = 2.817, P = 0.0005) has a slightly lower influence strength than PEV and CA but remains at a moderate driving level. ECP's f^2^ = 0.013, being a smaller effect size among BIP driving variables, suggests its unique explanatory power is relatively limited. However, it reflects the appeal of packaging's environmental attributes (e.g., degradable materials) to user groups with strong environmental awareness. Social cultural environment (SE) (β = 0.102, T = 2.456, P = 0.014) has the lowest path coefficient among all variables. SE's f^2^ = 0.011 is the smallest effect size among all significant paths. Despite reaching statistical significance (P = 0.014), its actual contribution is low. Although it passes the significance test, it more accurately reflects external auxiliary conditions (e.g., societal advocacy for “healthy eating,” subculture consumption trends) with a relatively limited direct driving effect on BIP.

Overall, the f^2^ values of BIP driving variables have not reached the medium effect threshold of 0.15, concentrating between 0.011 and 0.040. This result indicates that although each variable statistically significantly influences BIP, the unique explanatory power of any single variable for BIP is limited, and the formation of PRF purchase intention is more likely a consequence of synergistic effects among multiple variables.

Concurrently, a cross-sectional comparison of internal and external factors reveals that “product attribute external orientation” variables generally have greater influence than “internal factor orientation” variables. External variables directly related to product packaging, such as FPP and FIF, exhibit generally higher path coefficients than variables like CA and SE. From an effect size perspective, the average f^2^ of external variables (mean of FPP, FIF, PEV, ECP ≈ 0.023) is also higher than that of internal variables (mean of CA, SE, CIR ≈ 0.017), further supporting the conclusion that “external orientation variables contribute more to purchase intention.” Notably, among the influencing factors of social environment (SE), although the relationship is significant, its level of significance is low. This suggests that consumers’ purchase intentions for PRF are more significantly influenced by product packaging attributes. Consumers place greater emphasis on packaging, particularly the product's functional aspects, alignment with needs, and information transparency, rather than solely relying on the influence of the external social environment. Therefore, the packaging of PRF foods plays a crucial role in shaping consumer purchase intentions. This conclusion aligns with existing research highlighting the significant role of consumer perception of packaging in purchase intentions [34] and the prominent influence of packaging on consumers [90,91]. This provides clear practical guidance for PRF design and marketing, emphasizing the prioritization of optimizing packaging functionality and information delivery over excessive reliance on socio-cultural promotion, thereby providing a theoretical basis.

(2) The impact of variables on consumer healthy eating behavior (CHEB) demonstrates both path logic and intensity differences. The influence dimensions of CHEB exhibit a dual characteristic of “direct drive + indirect transmission,” with significant differences in impact intensity compared to the effect on purchase intention (BIP). This can be categorized into two main features: “chain transmission path” and “direct drive difference” and “core variable highlighting”: Consumer individual needs (CIR) have a significant positive impact on both BIP and CHEB, but with differing path coefficients. The impact on BIP (β = 0.158) is slightly higher than that on CHEB (β = 0.117, T = 2.718, P = 0.007). The effect size of CIR on CHEB was f^2^ = 0.013, and on BIP was f^2^ = 0.025. The former's proportion is substantially smaller than the latter's, further validating that CIR has a weaker influence on CHEB compared to BIP.This difference suggests a potential “indirect transmission” mechanism for the impact of CIR on CHEB. CIR first drives the formation of purchase intention by satisfying users’ core needs for PRF (such as nutrition and health attributes), and then, using purchase intention as an intermediary, further transmits to healthy eating behavior, forming a “CIR → BIP→CHEB” chain influence path. The effect size of BIP on CHEB was f^2^ = 0.033, significantly higher than the direct f^2^ (0.013) from CIR to CHEB. This corroborates the critical mediating role of purchase intention in the conversion from “individual needs” to “healthy behaviors.” This finding provides empirical support for understanding the “need-intention-behavior” transformation logic, indicating that consumers’ individual needs for PRF must be channeled through the “intermediary bridge” of purchase intention to more effectively translate into healthy eating behavior. Secondly, the differentiation of the role of packaging attribute variables shows that packaging environmental considerations (ECP) and perceived experiential value (PEV) have a greater impact on CHEB than packaging functional attributes (FPP). The path coefficient for ECP is 0.167 (T = 3.674, P = 0.000), and for PEV is 0.169 (T = 4.179, P = 0.000), both of which are close in value and significantly higher than the impact of FPP on CHEB (β = 0.101, T = 2.402, P = 0.016). In terms of effect size, ECP→CHEB had an f^2^ = 0.025, PEV→CHEB had an f^2^ = 0.029, and FPP→CHEB had an f^2^ = 0.010. Although the effect sizes of ECP and PEV are still considered small effects, they are approximately 2.5–3 times larger than that of FPP, consistent with the trend in path coefficient differences, further supporting that ECP and PEV have a significantly stronger driving effect on CHEB than FPP.This result indicates that, compared to FPP (focusing on the practical functions of packaging), ECP (packaging environmental friendliness) and PEV (user subjective experience) are more closely aligned with consumers’ core perceptual needs for healthy eating behavior. The former aligns with the cognition of “linking healthy eating with a sustainable lifestyle,” a view consistent with Kyung-A Sun's research on consumers’ perception of food healthiness [92]. The latter reinforces “the pleasure of healthy eating” through positive experiences, thereby more effectively promoting the formation of healthy eating behavior. The path coefficient of food information factors (FIF) to CHEB (β = 0.235) was the highest among all driving variables of CHEB. Its effect size, f^2^ = 0.054, was also the largest among the CHEB driving variables, significantly exceeding those of other variables (BIP's f^2^ = 0.033, PEV's f^2^ = 0.029, ECP's f^2^ = 0.025). Although f^2^ = 0.054 still represents a small effect, it is the highest value in this study's model, indicating that FIF possesses a relatively prominent unique explanatory power for CHEB and highlighting the central position of information factors in promoting the sustainability of healthy eating behaviors.

Based on the above discussion, stratifying the driving paths of consumer purchase intention (BIP) reveals that packaging functionality (FPP) is the key driving factor with the highest path coefficient. Its effect size, f^2^ = 0.040, although a small effect, is the largest among the BIP driving variables. This aligns with sensory marketing theory, which transforms abstract “perceived behavioral control” into concrete functional satisfaction, complementing relevant dimensions of TPB theory and echoing existing research findings. Concurrently, consumer individuality (CIR) and food information elements (FIF) exhibit similar path strengths and are significantly higher than attitude and socio-cultural environment. The effect sizes of CIR (f^2^ = 0.025) and FIF (f^2^ = 0.022) are also in the second tier of BIP driving variables, validating information processing theory, where precise information conveyed by packaging acts as a cognitive mediator for the “needs-intention” conversion, thereby filling a cognitive gap in TPB theory. Furthermore, the socio-cultural environment (SE) had the lowest path coefficient, and its f^2^ = 0.011 was the smallest among all significant paths. Although significant, its direct driving effect is limited, thus refining the application boundary of TPB theory in the context of PRF consumption, implying that PRF consumption is more focused on the match between product attributes and individual needs rather than social norm pressure.

Examining the differentiated impact paths on healthy eating behaviors (CHEB), the path coefficient of food information elements (FIF) was significantly higher than that of other variables, with an f^2^ = 0.054 also being the highest among CHEB driving variables. This is 5.4 times that of FPP (f^2^ = 0.010), with the effect size difference far exceeding the path coefficient difference. Moreover, it is considerably higher than its influence on purchase intention (BIP), validating the “depth of processing effect” from information processing theory. This not only affects short-term purchases but also solidifies behaviors through long-term cognitive penetration, promoting “single purchase-long-term consumption” and complementing the long-term influence mechanisms of integrated models of sensory marketing and TPB. The key mediating path: consumer purchase intention (BIP) significantly and positively influences consumer healthy eating behaviors (CHEB). The f^2^ for BIP→CHEB was 0.033, second only to FIF among CHEB driving variables, validating a core proposition of TPB theory. Furthermore, a chained transmission mechanism of “CIR → BIP→CHEB” exists, filling the mediating gap for the “individual needs→healthy behavior” conversion. Within the differentiated driving paths, eco-friendly packaging considerations (ECP) and perceived experiential value (PEV) had a significant impact on CHEB, exceeding that of packaging functional attributes (FPP). The effect sizes of ECP (f^2^ = 0.025) and PEV (f^2^ = 0.029) were both significantly higher than that of FPP (f^2^ = 0.010), supporting the integration logic of sensory marketing and sustainable consumption theories. ECP reinforces behavioral persistence through value identification, while PEV evokes positive emotions based on sensory experience, forming a “emotional attachment→behavioral persistence” pathway consistent with self-cognition theory. Overall, the f^2^ values of all significant paths in this study fall between 0.010 and 0.054, falling within the category of small effects [93]. This outcome is consistent with the expectations of exploratory research, suggesting that PRF consumption decisions are influenced by the synergy of multiple factors, with limited unique contributions from any single variable. Among these, FIF→CHEB (f^2^ = 0.054) and FPP → BIP (f^2^ = 0.040) were the two paths with relatively higher effect sizes, respectively highlighting the role of information factors in behavioral consolidation and the status of functional attributes in intention formation.

### Theoretical and practical contributions

#### Theoretical contributions.

This research focuses on the relationship between plant-based food (PRF) purchases and consumer healthy eating behaviors, with systematic innovation across three dimensions:

Firstly, regarding theoretical boundaries, I've moved beyond the traditional focus of the Theory of Planned Behavior (TPB) on consumer attitudes and subjective norms. I've innovatively integrated Information Processing Theory and Sensory Marketing Theory, incorporating variables like packaging attributes and perceived experiential value into the analytical framework. This study systematically reveals the synergistic logic between TPB's internal psychological variables and external product attribute variables for the first time. Through a mediation chain of “internal psychological motivation → external packaging stimulus → intention → behavior,” I've expanded the research application of the traditional TPB model, which previously emphasized internal psychology over external product attributes. This expansion not only elevates TPB from a “psychologically driven model” to a “dual-driven model of psychology and product” but also systematically unveils the specific driving mechanisms of “product packaging – information transmission – sensory experience” on PRF purchase intention (BIP) and healthy eating behavior (CHEB). This enriches the theoretical implications of TPB in the unique consumption scenario of plant-based foods and advances TPB from a “general behavior prediction model” to a “precision explanation model for segmented product scenarios,” overcoming its past limitations in food consumption research for neglecting the impact of concrete product attributes.

Secondly, in clarifying the mediating mechanisms, I've addressed the shortcomings of existing TPB research that treats “intention-behavior” as a singular linear relationship and lacks analysis of variable effect strength and path differences. This study quantimentatively validates the “hierarchical driving” characteristics of core variables for the first time. I've identified functional packaging attributes (FPP) as the strongest external driver of purchase intention (BIP) (β = 0.195), while environmental considerations of packaging (ECP) and perceived experiential value (PEV) are the core drivers of sustained healthy eating behavior (CHEB) (β = 0.167 and 0.169, respectively). Furthermore, consumer individual needs (CIR) can influence CHEB through a chained path of “CIR → BIP→CHEB.” This finding challenges the perception of “homogenized impact of variables on intention and behavior,” clearly delineating the functional differences of various variables across the entire chain of “intention formation – behavior conversion.” This advances TPB research from “verifying variable significance” to “analyzing differential variable effects,” surpassing the traditional TPB's focus solely on “the existence of variable associations” and providing empirical references for segmented dimensions in subsequent “intention-behavior” conversion studies.

Thirdly, in constructing the theoretical framework, I've addressed the fragmentation and lack of systematic integration in existing TPB application research. I've classified seven categories of core variables into “internal vs. external factors” and “intention drivers vs. behavior drivers,” thereby constructing a multi-dimensional linkage framework of “TPB core internal factors (consumer individual needs CIR, social environment SE, attitude CA) + external driving factors (product packaging elements FPP/ECP/FIF/PEV).” This framework not only validates the independent effects of each variable but also clarifies the interactive logic between external product attributes and TPB's internal variables (e.g., FIF indirectly influences BIP by strengthening CA, and PEV promotes CHEB by enhancing perceived behavioral control). This resolves the abstract nature of TPB's core constructs, building a complete loop of “external product attributes – internal psychological motivation – intention – behavior.” This integrated framework moves beyond the traditional TPB model of analyzing internal variables in isolation, offering a reusable paradigm for related cross-disciplinary research and providing innovative ideas for “multi-theory integration and multi-variable linkage” in the application of TPB to consumer behavior research. This advances TPB from a “single theoretical model” to a “cross-theoretical integrated framework.”

#### Practical contributions.

Based on the core conclusions revealed by the empirical research above, namely that packaging functional attributes are the strongest driver of PRF purchase intention, food information factors have a dual strong drive for achieving purchase intention and healthy eating behaviors, and packaging environmental considerations and perceived experiential value are the core drivers of healthy eating behaviors, I propose a three-dimensional comprehensive strategy combining industry pain points and policy needs: “Enterprise Precise Breakthrough + Industry Collaborative Upgrade + Policy Targeted Empowerment.” This strategy ensures a deep integration with empirical results and practical feasibility. At the enterprise level, focusing on core driving factors, efforts are concentrated on product and marketing. On the packaging front, an “function-information-environmental-experience” four-dimensional optimization system is established with FPP as the core. High-barrier materials are adopted to extend the shelf life of perishable PRFs, and modular portable designs are promoted to lower the consumption threshold. Food information factors involve constructing a dual information matrix of “decision-making and educational” types, quantifying core nutritional information, labeling trust marks, and supplementing long-term health benefits. Packaging environmental considerations and perceived experiential value are synergistically advanced through the application of degradable materials, complemented by naturalistic visual and gentle tactile designs to enhance sensory experience. The marketing side avoids the limitations of weak drivers from social and cultural environments, shifting to precise marketing based on “demand and scenario.” Product value is differentiated for fitness, environmental, and young consumer groups. A “packaging and scenario” linkage system is established, combining online and offline channels to amplify word-of-mouth effects and promote the conversion of purchase intention into healthy behaviors.

At the industry and policy levels, synergy and empowerment are strengthened based on the driving mechanisms to promote a full-chain upgrade and environmental optimization. On the industry side, industry associations will take the lead in integrating upstream and downstream resources to establish a centralized procurement platform for environmentally friendly functional materials and form a PRF packaging technology innovation alliance to tackle “high preservation and degradability” integrated technology, thereby reducing optimization costs for enterprises. Simultaneously, in conjunction with quality inspection institutions, the “Guideline for Labeling Information on PRF Product Packaging” will be formulated, clarifying mandatory labeling items and presentation standards for nutritional and environmental information to address information asymmetry. On the policy side, special subsidy policies will be introduced to provide food packaging cost subsidies for enterprises that use degradable materials and meet high preservation standards, and a technology innovation fund will be established to support SMEs in developing integrated packaging technologies. The “Mandatory Standard for PRF Nutritional Information Labeling” will be formulated to regulate information presentation, and concurrently, the “PRF Healthy Experience Popularization Project” will be launched. Through interpretation of packaging information in experience zones, taste tests, and the release of healthy eating guides, a closed-loop system of “policy popularization and packaging education” will be formed. This will foster a virtuous cycle among enterprises, industries, and policies, balancing scientific rigor with implementability, ultimately achieving the dual goals of increasing PRF industry penetration and popularizing healthy eating behaviors among consumers.

## Conclusion and suggestions

### Conclusion

This study, grounded in the Theory of Planned Behavior (TPB) and integrating Information Processing Theory and Sensory Marketing Theory, systematically investigates the mediating mechanisms through which internal and external leading variables of plant-based foods and their packaging influence consumers’ purchase intentions and healthy eating behaviors. Structural equation modeling analysis of 566 valid questionnaire responses yielded the following core conclusions:

This research achieves three breakthroughs: First, it transcends the traditional scope of TPB, which primarily focuses on internal psychological variables, by innovatively incorporating external product variables such as packaging functional attributes and food information factors into the analytical framework. This has resulted in a chained mediation model of “internal psychology-external stimuli-behavioral intention-healthy behavior,” advancing TPB from a “psychology-driven model” to a “psychology-product dual-driven model.” Second, by quantitatively comparing the path coefficients of various variables, it reveals for the first time the “hierarchical driving characteristics” of core variables on purchase intention (packaging functional attributes, β = 0.195, were strongest, while socio-cultural environment, β = 0.102, was weakest) and the “path differentiation logic” for healthy eating behaviors (packaging environmental considerations and perceived experiential value significantly outweighed packaging functional attributes). This surpasses the cognitive limitations of traditional research, which often assumes a homogenization of variable impacts on intentions and behaviors. Third, it constructs a multidimensional association framework integrating “core internal factors of TPB, external driving factors of product packaging,” clarifying the interactive mechanisms between external product attributes and internal psychological variables and providing a reusable theoretical paradigm for interdisciplinary research in the field of food packaging.

The research offers clear guidance for plant-based food companies and policymakers: First, packaging functional attributes are the strongest drivers of purchase intention (β = 0.195); companies should prioritize optimizing core functions such as preservation, portability, and ease of use, rather than over-relying on socio-cultural promotion. Second, food information factors significantly drive both purchase intention (β = 0.148) and healthy eating behaviors (β = 0.235); companies should establish a dual information matrix of “decision-making-oriented, educational,” quantifying core nutritional components and supplementing with information on long-term health benefits. Third, packaging environmental considerations (β = 0.167) and perceived experiential value (β = 0.169) are the core drivers for sustained healthy eating behaviors; companies should collaboratively promote the application of degradable materials and naturalist sensory design. Concurrently, policymakers can guide industry upgrades toward sustainability through specific subsidies and mandatory information labeling standards.

In summary, this study theoretically expands the application boundaries of TPB within the context of plant-based food consumption. Practically, it provides companies with a packaging design strategy centered on the “four-dimensional optimization of function-information-environment-experience” and offers policymakers empirical evidence and intervention pathways for promoting healthy and sustainable diets. Future research could further broaden sample coverage, adopt longitudinal tracking designs, and incorporate moderating variables such as cultural background to enhance the external validity and cross-cultural applicability of the findings.

### Suggestions

Regarding existing consumer research on food packaging, particularly concerning its health and environmental attributes, there remains room for improvement in sample representativeness and scenario adaptability, the dimensional measurement and dynamic tracking of variables, and the completeness of variable relationships. Concurrently, issues such as self-report bias, the limitations of cross-sectional designs, the neglect of cultural context, and online sampling biases warrant further attention. These shortcomings are manifested as follows:

Firstly, sample representativeness and scenario adaptability require further refinement. On one hand, current research inadequately covers the entire spectrum of age groups, geographical regions, and educational backgrounds. Young children and adolescents are often excluded, while the representation of middle-aged and elderly individuals (aged 55 and above, approximately 12.2%), those with lower educational attainment (around 23.3%), and rural populations is relatively limited. This deficiency hinders a comprehensive understanding of consumer perceptions, packaging interpretation abilities, and sensory sensitivities towards healthy and eco-friendly packaging (PRF). On the other hand, the inherent tendencies of online research channels lead to samples being concentrated among younger, digitally literate individuals, potentially introducing sampling biases that could affect the generalizability of research findings. Furthermore, existing studies have largely overlooked the differential impact of purchasing scenarios, with insufficient distinction made regarding variations in consumer needs for packaging functionalities and preferences for information acquisition channels across different contexts. Consequently, there is a lack of robust evidence for developing targeted design strategies.

Secondly, limitations also exist in variable measurement and research design. In terms of measurement dimensions, current research predominantly focuses on single sensory modalities when assessing perceived experiential value, with relatively insufficient systematic consideration given to multisensory integrated experiences. Regarding research design, the static analysis paradigm employing cross-sectional data is common, making it difficult to reveal the dynamic evolutionary patterns of inter-variable effects and challenging to track the long-term transition process of consumers from “purchase intention” to “healthy behavior.” Additionally, studies often rely heavily on self-reported consumer data, which can introduce a degree of self-report bias. For instance, respondents may embellish their behaviors due to social desirability bias or provide inaccurate data due to memory recall issues, thereby potentially compromising the reliability of research conclusions.

Thirdly, there is scope for enhancement in the completeness of variable relationships and cultural adaptability. Current research has not delved deeply enough into the complex mechanisms underlying the interplay between packaging attributes, consumer psychology, and behavior, while giving limited attention to the moderating role of cultural context. Consumers from different cultural backgrounds may hold varying value orientations towards healthy and eco-friendly packaging. However, existing studies have seldom incorporated cultural dimensions into their analytical frameworks, thus presenting certain limitations when extending these conclusions to cross-cultural contexts.

Based on the preceding analysis, future research can be enhanced in two primary aspects. Firstly, the sampling and research design require refinement. It is recommended to employ multistage stratified sampling to improve the representativeness of the sample concerning geographical location, age, education level, and income. Furthermore, “purchase scenario” should be utilized as a grouping variable to systematically compare the differential impacts of core variables across various scenarios. The integration of mixed-methods research, combining questionnaire surveys with objective behavioral data, is advised to mitigate the potential influence of self-reporting bias. Concurrently, cultural context should be incorporated as a moderator variable within the model to investigate packaging design strategies in cross-cultural settings. Secondly, the variable measurement and dynamic tracking system should be optimized. It is suggested to expand the measurement dimensions of perceived experiential value, thereby constructing a scale that encompasses the synergistic multisensory experience of visual, tactile, and olfactory modalities. The adoption of a longitudinal tracking design for long-term collection of consumer purchase intention and health-related dietary behavior change data will enable a more accurate elucidation of the dynamic mechanisms and critical influencing nodes in the “intention-behavior” conversion.

## References

[1] Emmanuella B, Ijeoma N, Sunday A, Ogo O, Ogbene I. Nutritional composition, bioactive compounds and antioxidant potential of pineapple rind flour as functional food ingredient. J Food Biochem. 2025;2025:8832878. doi: 10.1155/jfbc/8832878

[2] Crous-Bou M, Molinuevo JL, Sala-Vila A. Plant-rich dietary patterns, plant foods and nutrients, and telomere length. Advances in Nutrition. 2019;10:S296–303. doi: 10.1093/advances/nmz026 PMC6855941 31728493

[3] Hever J, Cronise RJ. Plant-based nutrition for healthcare professionals: implementing diet as a primary modality in the prevention and treatment of chronic disease. J Geriatr Cardiol. 2017;14(5):355–68. doi: 10.11909/j.issn.1671-5411.2017.05.012 28630615 PMC5466942

[4] Yu SJ, Morris A, Kayal A, Milošević I, Van TTH, Bajagai YS, et al. Pioneering gut health improvements in piglets with phytogenic feed additives. Appl Microbiol Biotechnol. 2024;108(1):142. doi: 10.1007/s00253-023-12925-2 38231265 PMC10794284

[5] Waheed Janabi AH, Kamboh AA, Saeed M, Xiaoyu L, BiBi J, Majeed F, et al. Flavonoid-rich foods (FRF): A promising nutraceutical approach against lifespan-shortening diseases. Iran J Basic Med Sci. 2020;23(2):140–53. doi: 10.22038/IJBMS.2019.35125.8353 32405356 PMC7211351

[6] Aboussaleh Y, Capone R, Bilali HE. Mediterranean food consumption patterns: low environmental impacts and significant health-nutrition benefits. Proc Nutr Soc. 2017;76(4):543–8. doi: 10.1017/S0029665117001033 28659225

[7] Trolle E, Nordman M, Lassen AD, Colley TA, Mogensen L. Carbon Footprint Reduction by Transitioning to a Diet Consistent with the Danish Climate-Friendly Dietary Guidelines: A Comparison of Different Carbon Footprint Databases. Foods. 2022;11(8):1119. doi: 10.3390/foods11081119 35454705 PMC9030092

[8] Kim H, Caulfield LE, Garcia-Larsen V, Steffen LM, Coresh J, Rebholz CM. Plant-Based Diets Are Associated With a Lower Risk of Incident Cardiovascular Disease, Cardiovascular Disease Mortality, and All-Cause Mortality in a General Population of Middle-Aged Adults. J Am Heart Assoc. 2019;8(16):e012865. doi: 10.1161/JAHA.119.012865 31387433 PMC6759882

[9] Consavage Stanley K, Hedrick VE, Serrano E, Holz A, Kraak VI. US Adults’ Perceptions, Beliefs, and Behaviors towards Plant-Rich Dietary Patterns and Practices: International Food Information Council Food and Health Survey Insights, 2012-2022. Nutrients. 2023;15(23). doi: 10.3390/nu15234990 PMC10708400 38068852

[10] Anjos O, Pires PCP, Gonçalves J, Estevinho LM, Mendonça AG, Guiné RPF.Plant-Based Beverages: Consumption Habits, Perception and Knowledge on a Sample of Portuguese Citizens. Foods. 2024;13:3235. 39456297 10.3390/foods13203235 PMC11507175

[11] Anwar RS, Ahmed RR, Streimikiene D, Streimikis J, Zamek D. Securing food futures: the interplay of safety governance, hygiene, supplier beliefs and consumer engagement. British Food Journal. 2024;127(9):758–78. doi: 10.1108/BFJ-09-2024-0882

[12] Ajzen I. The theory of planned behavior. Organizational Behavior and Human Decision Processes. 1991;50:179–211. doi: 10.1016/0749-5978(91)90020-T

[13] Petty RE, Cacioppo JT. The elaboration likelihood model of persuasion. Communication and persuasion: Central and peripheral routes to attitude change. New York, NY: Springer New York. 1986. 1–24.

[14] Krishna A. An integrative review of sensory marketing: Engaging the senses to affect perception, judgment and behavior. Journal of Consumer Psychology. 2012;22:332–51. doi: 10.1016/j.jcps.2011.08.003

[15] Tavares Filho ER, Silva R, Campelo PH, Platz VHCB, Spers EE, Freitas MQ, et al. Think and Choose! The Dual Impact of Label Information and Consumer Attitudes on the Choice of a Plant-Based Analog. Foods. 2024;13(14):2269. doi: 10.3390/foods13142269 39063353 PMC11275425

[16] Abidin SZ, Warell A, Liem A. The significance of form elements: a study of representational content of design sketches. In: Proceedings of the Second Conference on Creativity and Innovation in Design, Eindhoven, Netherlands, 2011. 21–30.

[17] Hassan ZZA, Shahriman, Anwar R, Vermol VV. The value of unintended human behaviour in everyday product design. In: Proceedings of the DS 117: Proceedings of the 24th International Conference on Engineering and Product Design Education (E&PDE 2022), London South Bank University, London, UK, 2022.

[18] Godin G, Kok G. The theory of planned behavior: a review of its applications to health-related behaviors. Am J Health Promot. 1996;11(2):87–98. doi: 10.4278/0890-1171-11.2.87 10163601

[19] Reina Paz MD, Rodríguez Vargas JC. Main theoretical consumer behavioural models. A review from 1935 to 2021. Heliyon. 2023;9(3):e13895. doi: 10.1016/j.heliyon.2023.e13895 36915520 PMC10006455

[20] Ramanankonenana T, Randriamamonjy L. Effect of sensory marketing in consumer behavior during the act of purchase: a case study of supermarkets. International Journal of Economics and Business Issues. 2023;2(2):13–21. doi: 10.59092/ijebi.vol2.Iss2.32

[21] Simmonds G, Spence C. Thinking inside the box: How seeing products on, or through, the packaging influences consumer perceptions and purchase behaviour. Food Quality and Preference. 2017;62:340–51. doi: 10.1016/j.foodqual.2016.11.010

[22] Spence C, Van Doorn G. Visual communication via the design of food and beverage packaging. Cogn Res Princ Implic. 2022;7(1):42. doi: 10.1186/s41235-022-00391-9 35551542 PMC9098755

[23] Rini L, Schouteten JJ, Faber I, Bechtold K-B, Perez-Cueto FJA, Gellynck X, et al. Identifying the Key Success Factors of Plant-Based Food Brands in Europe. Sustainability. 2022;15(1):306. doi: 10.3390/su15010306

[24] Chinburapa V, Larson LN, Brucks M, Draugalis J, Bootman JL, Puto CP. Physician prescribing decisions: the effects of situational involvement and task complexity on information acquisition and decision making. Soc Sci Med. 1993;36(11):1473–82. doi: 10.1016/0277-9536(93)90389-l 8511635

[25] Hussain S. Brand Image and Customer Loyalty Through Sensory Marketing Strategies - A Study on International Fast Food Chain Restaurants. ijms. 2018;V(2(7)):32. doi: 10.18843/ijms/v5i2(7)/05

[26] Suurmets S, Clement J, Piras S, Barlagne C, Tura M, Mokhtari N, et al. Utilizing Sensory and Visual Data in the Value Estimation of Extra Virgin Olive Oil. Foods. 2024;13(18):2904. doi: 10.3390/foods13182904 39335835 PMC11430995

[27] Giampietri E, Verneau F, Del Giudice T, Carfora V, Finco A. A Theory of Planned Behaviour Perspective for Investigating the Role of Trust in Consumer Purchasing Decision Related to Short Food Supply Chains. Food Quality and Preference. 2018;64:160–6. doi: 10.1016/j.foodqual.2017.09.012

[28] Bukhari F, Hussain S, Ahmed RR, Mubasher KA, Naseem MR, Rizwanullah M, et al. Consumers’ purchase decision in the context of western imported food products: Empirical evidence from Pakistan. Heliyon. 2023;9(10):e20358. doi: 10.1016/j.heliyon.2023.e20358 37771538 PMC10522991

[29] Armitage CJ, Conner M. Efficacy of the Theory of Planned Behaviour: a meta-analytic review. Br J Soc Psychol. 2001;40(Pt 4):471–99. doi: 10.1348/014466601164939 11795063

[30] Ayar I, Gürbüz A. Sustainable consumption intentions of consumers in Turkey: A research within the theory of planned behavior. SAGE Open. 2021;11. doi: 10.1177/21582440211047563

[31] Donahue ME. Theory of Planned Behavior Analysis and Organic Food Consumption of American Consumers. 2017.

[32] Fauzi MA, Hasan MN, Zulkepeli L, Karuppiah K. Organic food consumption and the theory of planned behaviour: science mapping of present and future trends. British Food Journal. 2025;127(8):2741–58. doi: 10.1108/bfj-09-2024-0931

[33] Guillard V, Gaucel S, Fornaciari C, Angellier-Coussy H, Buche P, Gontard N. The Next Generation of Sustainable Food Packaging to Preserve Our Environment in a Circular Economy Context. Front Nutr. 2018;5:121. doi: 10.3389/fnut.2018.00121 30564581 PMC6288173

[34] Silayoi P, Speece M. The importance of packaging attributes: a conjoint analysis approach. European Journal of Marketing. 2007;41(11/12):1495–517. doi: 10.1108/03090560710821279

[35] Broucke J, Noeth E, Du Bois E. Vegetables and fruits in a circular economy: packaging challenges and design opportunities. Proc Des Soc. 2023;3:3781–90. doi: 10.1017/pds.2023.379

[36] Prabowo P, Soekardi C. SMEs’ Eco-Friendly Food Packaging Critical Factors: An Empirical Study. ijaste. 2023;1(2):529–35. doi: 10.24912/ijaste.v1.i2.529-535

[37] Arboretti R, Bordignon P. Consumer preferences in food packaging: CUB models and conjoint analysis. British Food Journal. 2016;118:527–40. doi: 10.1108/BFJ-04-2015-0146

[38] Francis DV, Dahiya D, Gokhale T, Nigam PS. Sustainable packaging materials for fermented probiotic dairy or non-dairy food and beverage products: challenges and innovations. AIMS Microbiol. 2024;10(2):320–39. doi: 10.3934/microbiol.2024017 38919715 PMC11194616

[39] Katz D. The Functional Approach to the Study of Attitudes. Public Opinion Quarterly. 1960;24(2, Special Issue: Attitude Change):163. doi: 10.1086/266945

[40] Latané B. The psychology of social impact. American Psychologist. 1981;36(4):343–56. doi: 10.1037/0003-066x.36.4.343

[41] Bandura A. Self-efficacy: toward a unifying theory of behavioral change. Psychol Rev. 1977;84(2):191–215. doi: 10.1037//0033-295x.84.2.191 847061

[42] Barsalou LW. Grounded cognition. Annu Rev Psychol. 2008;59:617–45. doi: 10.1146/annurev.psych.59.103006.093639 17705682

[43] Barbosa SC, Matteucci MBA, Leandro WM, Leite AF, Cavalcante ÉLS, Almeida GQE. Perfil do consumidor e oscilações de preços de produtos agroecológicos. Pesquisa Agropecuária Tropical. 2011;41:602–9.

[44] Czudec A, Miś T, Zając D. Supporting local economic development as a motive for purchasing organic food. Ekonomia i Środowisko - Economics and Environment. 2022;81:291–312. doi: 10.34659/eis.2022.81.2.444

[45] Park CI, Namkung Y. Consumer values, attitudes, and behavior towards plant-based alternatives. 2024;13:2561. 10.3390/foods13162561 PMC11353567 39200488

[46] Muhammad SA. Influence of family, peers, and media on children’s participation in the family buying process. Review of Education, Administration and Law (REAL). 2022;5(3):489–98. doi: 10.47067/real.v5i3.278

[47] Karimah F, Nurdin N, Hestiningtyas W. The Effect of Social Influence on Students’ Purchase Decision at Marketplace of Shopee. Journal of Economics Education and Entrepreneurship. 2022;3(1):65. doi: 10.20527/jee.v3i1.4309

[48] Lassen AD, Christensen LM, Trolle E. Development of a Danish Adapted Healthy Plant-Based Diet Based on the EAT-Lancet Reference Diet. Nutrients. 2020;12(3):738. doi: 10.3390/nu12030738 32168838 PMC7146415

[49] Pieszak E. The Culture of Consumption as a Consequence of the Economic Process in the Context of Changes in the European Union. 1014746/rie. 2020;(14):259–69. doi: 10.14746/rie.2020.14.17

[50] Ryan RM, Deci EL. Self-determination theory and the facilitation of intrinsic motivation, social development, and well-being. Am Psychol. 2000;55(1):68–78. doi: 10.1037//0003-066x.55.1.68 11392867

[51] Albornoz R, García-Salirrosas EE, Millones-Liza DY, Villar-Guevara M, Toyohama-Pocco G. Using the Theory of Perceived Value to Determine the Willingness to Consume Foods from a Healthy Brand: The Role of Health Consciousness. Nutrients. 2024;16(13):1995. doi: 10.3390/nu16131995 38999743 PMC11243000

[52] Erciş A, Deveci FG, T Y TY. Investigation of consumers’ organic food purchases in the context of the relationship of personal values and individual factors. Mehmet Akif Ersoy University-Journal of Economics and Administrative Sciences Faculty. 2020;7:297–325. doi: 10.30798/makuiibf.573331

[53] Anh P, Hoang M. Determinants impacting consumers’ purchase intention: The case of fast food in Vietnam. International Journal of Marketing Studies. 2016;8(5):56. doi: 10.5539/ijms.v8n5p56

[54] Wang J, Pham TL, Dang VT. Environmental consciousness and organic food purchase intention: A moderated mediation model of perceived food quality and price sensitivity. Sustainability. 2020;17(850). 10.3390/ijerph17030850 PMC7037607 32013260

[55] Ms M. Perceived price and organic food consumption behaviour. 2021.

[56] Wangpo K, Wangmo S. The influence of social media marketing on purchase intention: The mediating role of brand equity. Asian Journal of Research in Marketing. 2022;11:20–33. doi: 10.5958/2277-6621.2022.00018.4

[57] Phuong N, Nguyen L. A study on game consumer behavior. Management Science Letters. 2021;11:2323–30. doi: 10.5267/j.msl.2021.6.002

[58] Dinu M, Abbate R, Gensini GF, Casini A, Sofi F. Vegetarian, vegan diets and multiple health outcomes: A systematic review with meta-analysis of observational studies. Crit Rev Food Sci Nutr. 2017;57(17):3640–9. doi: 10.1080/10408398.2016.1138447 26853923

[59] Satija A, Bhupathiraju SN, Rimm EB, Spiegelman D, Chiuve SE, Borgi L, et al. Plant-Based Dietary Patterns and Incidence of Type 2 Diabetes in US Men and Women: Results from Three Prospective Cohort Studies. PLOS Medicine. 2016;13:e1002039. doi: 10.1371/journal.pmed.1002039 PMC4907448 27299701

[60] Sottile F, Massaglia S, Merlino VM, Peano C, Mastromonaco G, Fornara F, et al. Consumption <i>vs</i>. non-consumption of plant-based beverages: A case study on factors influencing consumers’ choices. AIMSAGRI. 2023;8(3):889–913. doi: 10.3934/agrfood.2023047

[61] Azad N, Hamdavipour L. A study on effects of packaging characteristics on consumer’s purchasing confidence. MSL. 2012;2(1):397–402. doi: 10.5267/j.msl.2011.07.004

[62] Sparsh Rathi DRR. A study on the effect of product packaging elements on consumer buying behaviour. IJDR. 2023;:63754–61. doi: 10.37118/ijdr.27170.09.2023

[63] Yeo SF, Tan CL, Lim KB, Khoo YH. Product packaging: Impact on customers’ purchase intention. International Journal of Business and Society. 2020;21:857–64. doi: 10.33736/ijbs.3298.2020

[64] Hersey JC, Wohlgenant KC, Arsenault JE, Kosa KM, Muth MK. Effects of front-of-package and shelf nutrition labeling systems on consumers. Nutr Rev. 2013;71(1):1–14. doi: 10.1111/nure.12000 23282247

[65] Van der Laan LN, De Ridder DTD, Viergever MA, Smeets PAM. Appearance matters: neural correlates of food choice and packaging aesthetics. PLoS One. 2012;7(7):e41738. doi: 10.1371/journal.pone.0041738 22848586 PMC3404976

[66] Chu R, Tang T, Hetherington MM. The impact of packaging attributes on portion decisions: Consumer values are important. Nutr Bull. 2024;49(3):314–26. doi: 10.1111/nbu.12688 38845598

[67] Ahmed R, Streimikiene D, Channar Z, Soomro DR, Streimikis J. E-banking customer satisfaction and loyalty: evidence from serial mediation through modified e-S-QUAL model and second-order PLS-SEM. Engineering Economics. 2021;32:407–21. doi: 10.5755/j01.ee.32.5.28997

[68] Jackson DL. Revisiting sample size and number of parameter estimates: Some support for the N:q hypothesis. Structural Equation Modeling: A Multidisciplinary Journal. 2003;10:128–41.

[69] Manterola C, Grande L, Otzen T, García N, Salazar P, Quiroz G. Confiabilidad, precisión o reproducibilidad de las mediciones. Métodos de valoración, utilidad y aplicaciones en la práctica clínica. Revista chilena de infectología. 2018;35:680–8. 31095189 10.4067/S0716-10182018000600680

[70] Lisawadi S, Ahmed SE, Reangsephet O, Shah MKA. Simultaneous estimation of Cronbach’s alpha coefficients. Communications in Statistics - Theory and Methods. 2019;48:3236–57. doi: 10.1080/03610926.2018.1473882

[71] Willis CE, Perlack RD. Multicollinearity: Effects, Symptoms, and Remedies. Journal of the Northeastern Agricultural Economics Council. 1978;7:55–61. doi: 10.1017/S0163548400001989

[72] Alhamami M, Alduais A, Qasem F, Alasmari M. Psychometric Features of the Arabic Version of the Children’s Communication Checklist (CCC2). J Multidiscip Healthc. 2024;17:3247–64. doi: 10.2147/JMDH.S462462 39006871 PMC11246085

[73] Bria M, Djakfar L, Wicaksono A. The impacts of mediating the work environment on the mode choice in work trips. Open Engineering. 2021;11:592–605. doi: 10.1515/eng-2021-0058

[74] Gorsuch RL. Using Bartlett’s significance test to determine the number of factors to extract. Educ Psychol Meas. 1973;33:361–4. doi: 10.1177/001316447303300216

[75] Tobias S, Carlson JE. Brief report: bartlett’s test of sphericity and chance findings in factor analysis. Multivariate Behav Res. 1969;4(3):375–7. doi: 10.1207/s15327906mbr0403_8 26745847

[76] Samiuddin M, Hanif M, Asad H. Some comparisons of the Bartlett and cube root tests of homogeneity of variance. Biometrika. 1978;65(1):218–21. doi: 10.1093/biomet/65.1.218

[77] Kashiwagi S. A new proposition on number of factors and communalities in multiple-factor analysis. Japanese Psychological Research. 1964;6:173–5. doi: 10.4992/psycholres1954.6.173

[78] Schmitt TA, Sass DA, Chappelle W, Thompson W. Selecting the “Best” Factor Structure and Moving Measurement Validation Forward: An Illustration. J Pers Assess. 2018;100(4):345–62. doi: 10.1080/00223891.2018.1449116 29630411

[79] Churchill GA. A paradigm for developing better measures of marketing constructs. Journal of Marketing Research. 1979;16:64–73. doi: 10.2307/3150876

[80] Fornell C, Larcker David F. Evaluating structural equation models with unobservable variables and measurement error. Journal of Marketing Research. 1981;18:39–50.

[81] Leguina A. A primer on partial least squares structural equation modeling (PLS-SEM). International Journal of Research & Method in Education. 2015;38(2):220–1. doi: 10.1080/1743727x.2015.1005806

[82] Henseler J, Ringle CM, Sarstedt M. A new criterion for assessing discriminant validity in variance-based structural equation modeling. J of the Acad Mark Sci. 2014;43(1):115–35. doi: 10.1007/s11747-014-0403-8

[83] Hair J, Hult GTM, Ringle C, Sarstedt M. A primer on partial least squares structural equation modeling (PLS-SEM). Thousand Oaks, CA, USA: SAGE Publications, Inc. 2014.

[84] Wetzels M, Odekerken-Schröder G, van Oppen C. Using PLS Path Modeling for Assessing Hierarchical Construct Models: Guidelines and Empirical Illustration1. MIS Quarterly. 2009;33(1):177–95. doi: 10.2307/20650284

[85] Shi D, Maydeu-Olivares A, Rosseel Y. Assessing Fit in Ordinal Factor Analysis Models: SRMR vs. RMSEA. Structural Equation Modeling: A Multidisciplinary Journal. 2020;27:1–15. doi: 10.1080/10705511.2019.1611434

[86] Ximénez C, Maydeu-Olivares A, Shi D, Revuelta J. Assessing Cutoff Values of SEM Fit Indices: Advantages of the Unbiased SRMR Index and Its Cutoff Criterion Based on Communality. Structural Equation Modeling: A Multidisciplinary Journal. 2022;29:368–80. doi: 10.1080/10705511.2021.1992596

[87] Bader M, Moshagen M. Assessing the fitting propensity of factor models. Psychol Methods. 2025;30(2):254–70. doi: 10.1037/met0000529 36201821

[88] Japutra A, Kumar Roy S, Pham TAN. Relating brand anxiety, brand hatred and obsess: moderating role of age and brand affection. Journal of Retailing and Consumer Services. 2021;60:102465. doi: 10.1016/j.jretconser.2021.102465

[89] Gowen AA, Downey G, Esquerre C, O’Donnell CP. Preventing over‐fitting in PLS calibration models of near‐infrared (NIR) spectroscopy data using regression coefficients. Journal of Chemometrics. 2010;25(7):375–81. doi: 10.1002/cem.1349

[90] Ampuero O, Vila N. Consumer perceptions of product packaging. Journal of Consumer Marketing. 2006;23(2):100–12. doi: 10.1108/07363760610655032

[91] Underwood RL. The communicative power of product packaging: Creating brand identity via lived and mediated experience. Journal of Marketing Theory and Practice. 2003;11:62–76. doi: 10.1080/10696679.2003.11501933

[92] Sun K-A, Moon J. The Relationship between Food Healthiness, Trust, and the Intention to Reuse Food Delivery Apps: The Moderating Role of Eco-Friendly Packaging. Foods. 2024;13(6):890. doi: 10.3390/foods13060890 38540879 PMC10969574

[93] Cohen J. Statistical power analysis for the behavioral sciences. 2nd ed. ed. New York: Routledge. 1988.

